# Supplementary material for: Exploring the role of readthrough-inducing molecule 2,6-diaminopurine to increase immune response against cancer cells
Source: Mol Ther. 2025 Sep 12;33(12):6212–25. doi: 10.1016/j.ymthe.2025.09.024 (PMC12703180; doi:10.1016/j.ymthe.2025.09.024)
Supplement: Document S1. Figures S1–S5 and supplemental method [file mmc1.pdf]

YMTHE, Volume *33*

Supplemental Information

Exploring the role of readthrough-inducing molecule 2,6-diaminopurine to increase immune response against cancer cells

Carmen Sandoval Pacheco, Alice M. Leroy, Mehdi Derhourhi, Tristan Cardon, Catherine Leroy, Nathalie Jouy, Emmanuelle Com, Blandine Guevel, Roland Bourette, Julie Carrard, Daniela Barros, Belinda Duchêne, Bénédicte Toussaint, Philippe Froguel, Nicolas Jonckheere, Thierry Chassat, Isabelle Van Seuningen, Régis Lavigne, Charles Pineau, Philippe Pierre, Fabrice Soncin, Michel Salzet, Amélie Bonnefond, and Fabrice Lejeune

Supplemental information

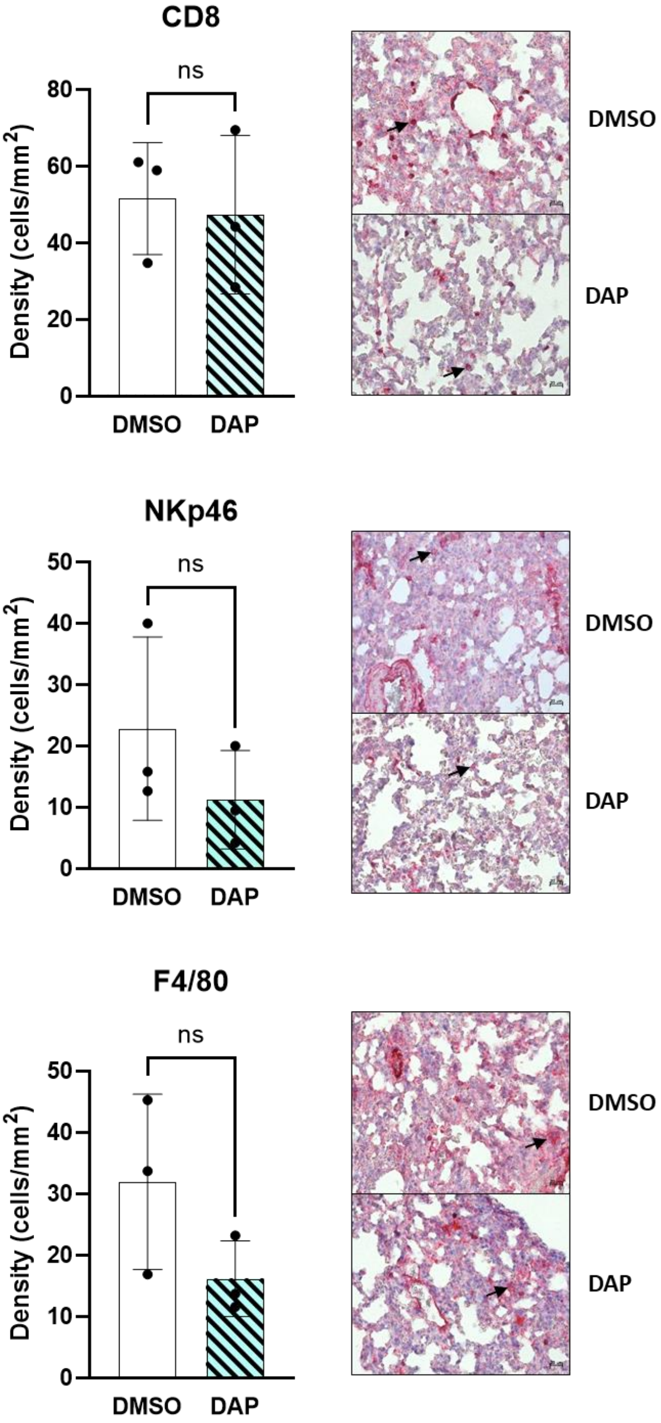

Figure S1: immunohistochemistry analysis of lymphocytes CD8+ (CD8, upper), natural killer (middle, NKp46) and macrophage (lower, F4/80) cells in healthy lung tissue after mouse treatment with DMSO or DAP. On the left is shown the quantification from 3 tumor slides. One representative picture of the analysis is presented on the right. Black arrows indicate the expression of interest. P-values were calculated by T-Test: n.s: statistically not significant.

A

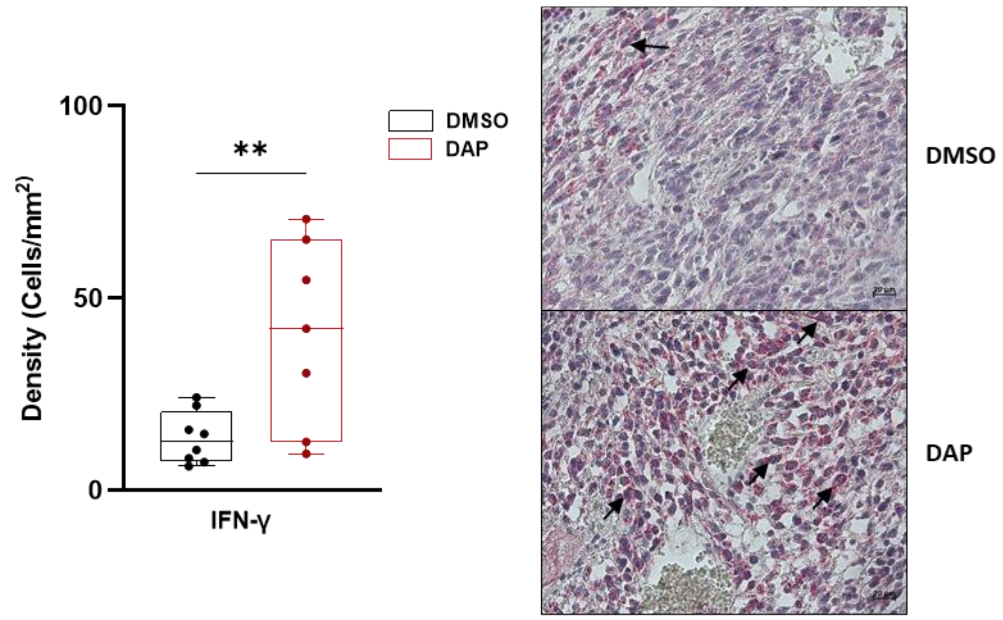

B

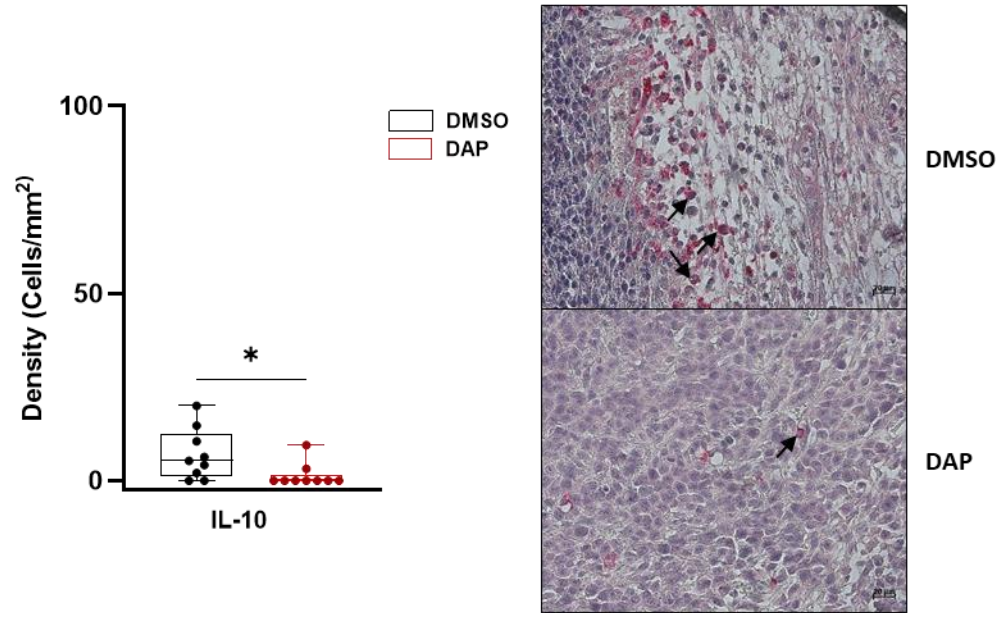

Figure S2: Immunohistochemistry analysis of IFN-γ expression (upper panel) and IL-10 expression (lower panel) in mice treated with DMSO or DAP. On the left is shown the quantification from 10 tumor slides. One representative picture of the analysis is presented on the right. Black arrows indicate the expression of interest. P-values were calculated by T-Test *<0.05; **<0.01.

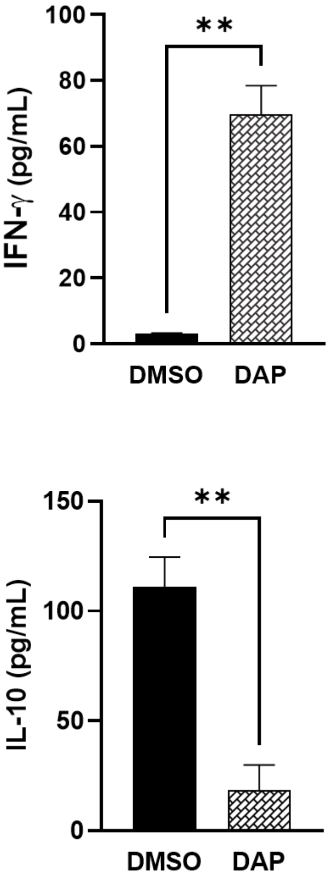

Figure S3: Quantification of IFN-γ and IL-10 expression by ELISA in co-cultures of CT26 cells and white blood cells (WBCs) isolated from the spleen of an untreated Balb/C mouse. WBCs and CT26 cells were co-cultured for 24 hours in the presence of anti-CD3 and anti-CD28 antibodies and either DMSO or DAP, followed by ELISA-based measurement of IFN-γ and IL-10 levels. P-values were calculated using a T-test **$p < 0.01$. The results shown are representative of two independent experiments.

First tumor

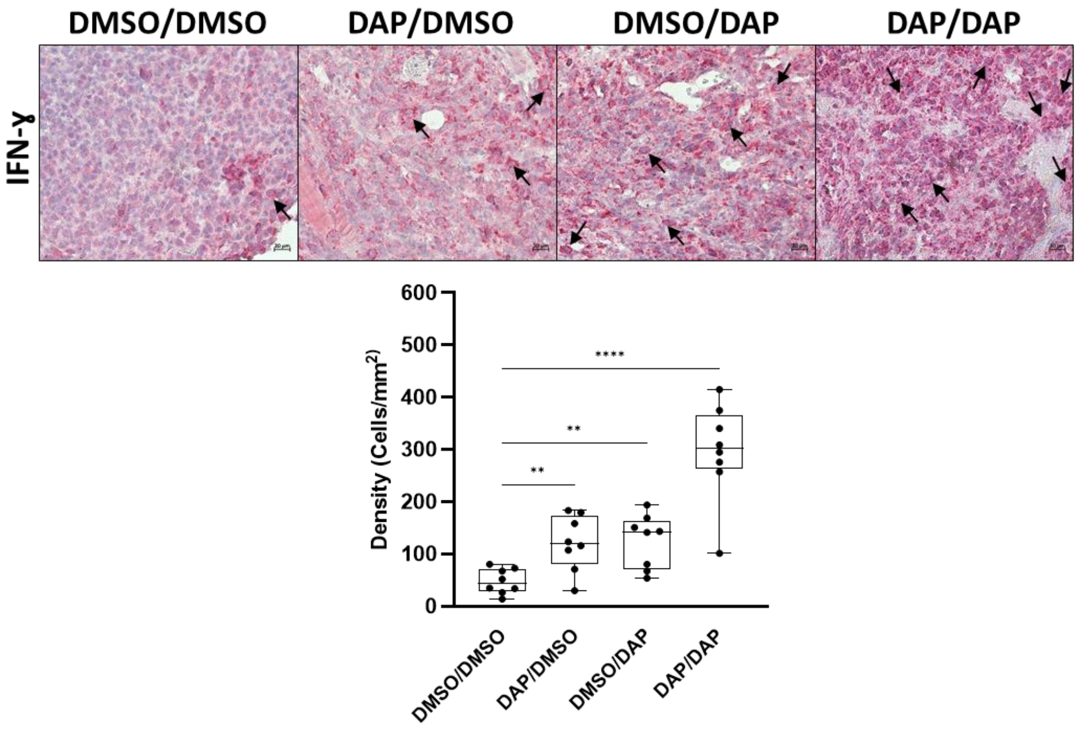

Second tumor

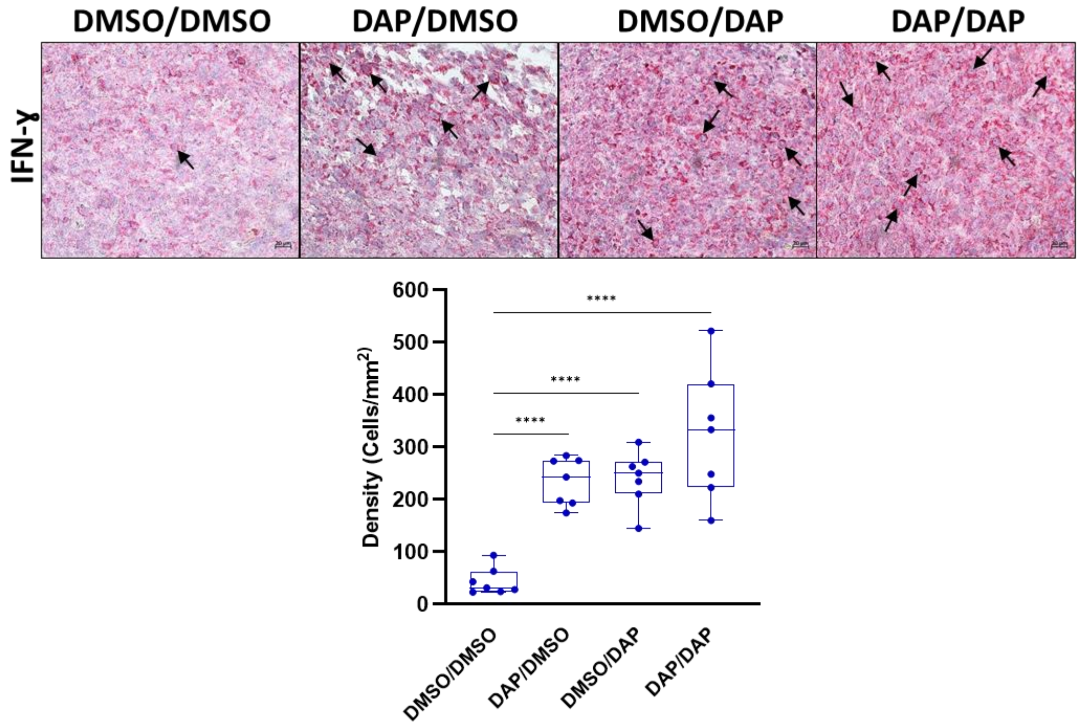

Figure S4: immunohistochemistry analysis of IFN-γ expression in first tumors (upper panel) and second tumors (lower panel) issued from cells treated with DMSO or 100 µM DAP. Quantification is based on the analysis of 10 pictures. One representative picture of the analysis is presented on the right. The

black arrows indicate the expression of interest. P-values were calculated by T-Test *<0.05; **<0.01; ***<0.001; ****<0.0001.

First Tumor

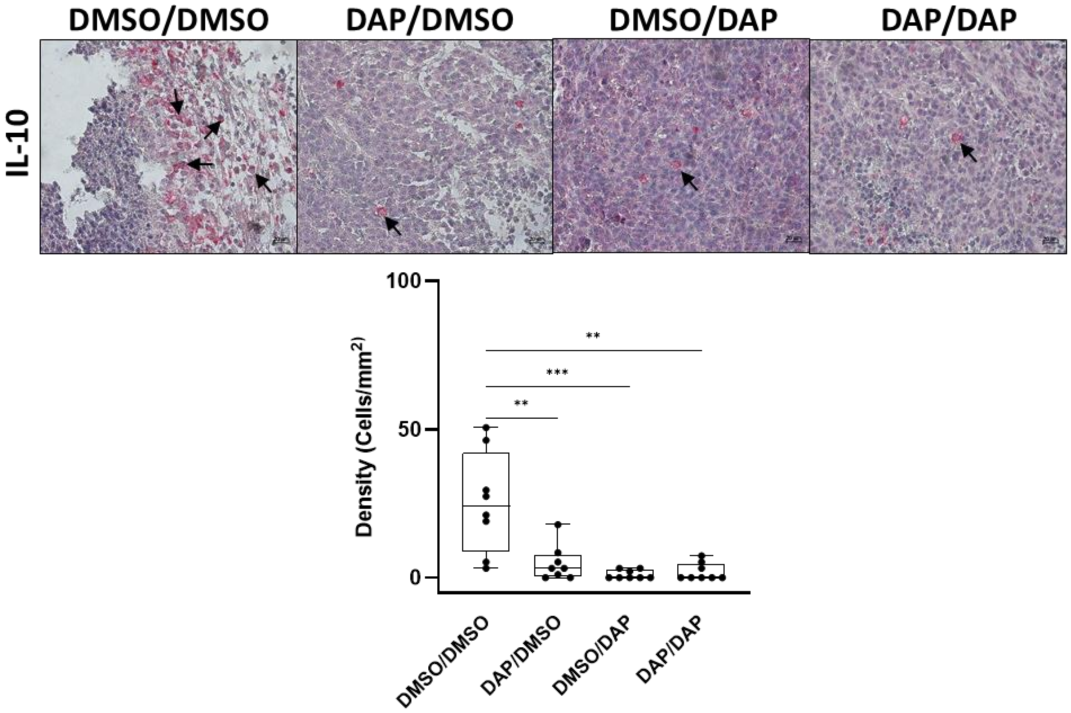

Second Tumor

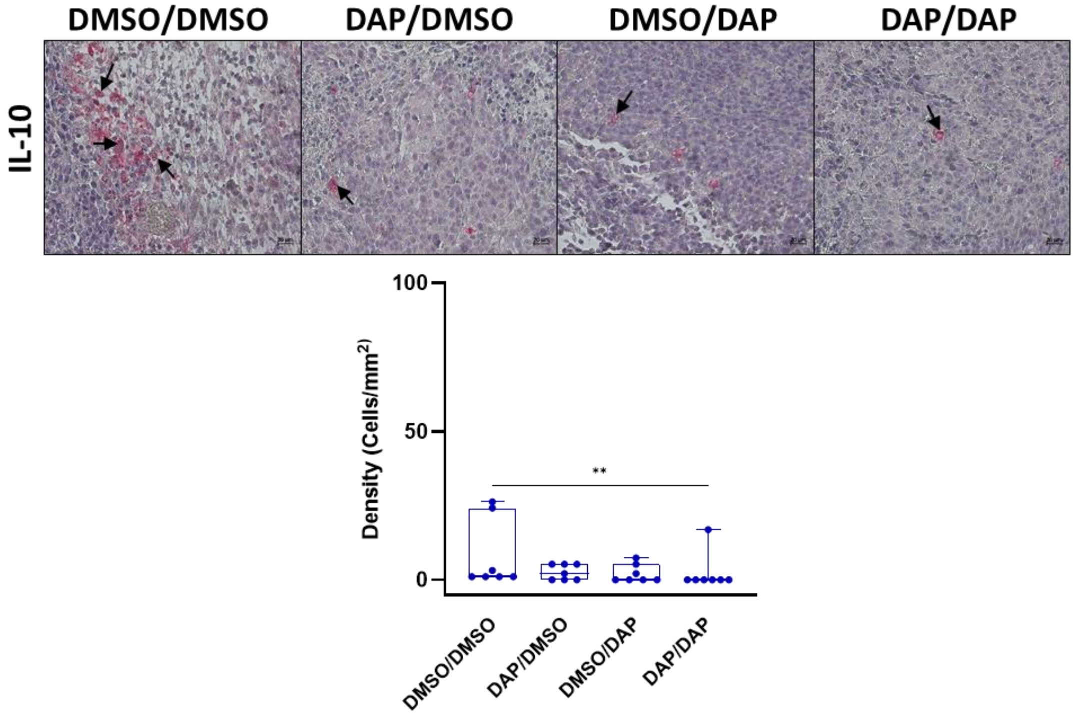

Figure S5: immunohistochemistry analysis of interleukin 10 expression in first tumors (upper panel) and second tumors (lower panel) issued from cells treated with DMSO or 100 µM DAP. Quantification

is based on the analysis of 10 pictures. One representative picture of the analysis is presented on the right. Black arrows indicate the expression of interest. P-values were calculated by T-Test *<0.05; **<0.01; ***<0.001; ****<0.0001.

Table S1: list of proteins that have their expression modified in the presence of DAP compared to DMSO treatment.

Supplemental method

Isolation of White Blood Cells (WBC) from Mouse Spleen

Splenic white blood cells were collected from BALB/c mice. We used a 70 µm cell strainer for mechanical dissociation into cold PBS. Red blood cells were removed using hypotonic in-house lysis buffer containing 155 mM NH_4Cl, 10 mM $KHCO_3$, and 0.1 mM EDTA at pH 7.4. After a 5-minute incubation, the cells were washed with 10 mL of PBS and then resuspended in complete RPMI medium containing 10% fetal bovine serum (Sigma-Aldrich) and 1% zellshield (Minerva Biolabs).

In vitro CT26 cells and WBC co-culture assay

CT26 cells were seeded in 24-well plates at a density of 1×10^5 cells per well and allowed to adhere overnight. Cells were then treated for 24 hours with either DMSO or 25µM DAP. Following the treatment, WBCs were added at a 5:1 ratio (WBC /tumor cell). T cell activation was performed by adding anti-CD3 (ref 16-0032-82, Invitrogen) and anti-CD28 (ref 16-0281-82, Invitrogen) at a 1:3 ratio. Cocultures were incubated at 37°C with 5% CO_2 for 24 hours. The supernatants were harvested, and the IL-10 and IFN-γ levels were measured.

ELISA

Concentrations of IFN-ɣ and IL-10 in cell culture supernatants were determined using commercially available ELISA kits for mouse cytokines (BMS606-2 and BMS614, Invitrogen), respectively, according to the manufacturer’s instructions. Absorbance was measured at 450 nm.
