# Supplementary material for: Exploring the role of readthrough-inducing molecule 2,6-diaminopurine to increase immune response against cancer cells
Source: Mol Ther. 2025 Sep 12;33(12):6212–25. doi: 10.1016/j.ymthe.2025.09.024 (PMC12703180; doi:10.1016/j.ymthe.2025.09.024)
Supplement: Document S2. Article plus supplemental information [file mmc3.pdf]

Molecular Therapy
Original Article

Exploring the role of readthrough-inducing molecule 2,6-diaminopurine to increase immune response against cancer cells

Carmen Sandoval Pacheco,[1] Alice M. Leroy,[2,3] Mehdi Derhourhi,[4] Tristan Cardon,[5] Catherine Leroy,[1] Nathalie Jouy,[6] Emmanuelle Com,[7,8] Blandine Guevel,[7,8] Roland Bourette,[1] Julie Carrard,[1] Daniela Barros,[9,10] Belinda Duchêne,[1] Bénédicte Toussaint,[4] Philippe Froguel,[4,11] Nicolas Jonckheere,[1] Thierry Chassat,[12] Isabelle Van Seuningen,[1] Régis Lavigne,[7,8] Charles Pineau,[7,8] Philippe Pierre,[9,10,13] Fabrice Soncin,[2,3] Michel Salzet,[5,14] Amélie Bonnefond,[4,11] and Fabrice Lejeune[1]

[1]University Lille, CNRS, INSERM, UMR9020-U1277 - CANTHER - Cancer Heterogeneity Plasticity and Resistance to Therapies, 59000 Lille, France; [2]CNRS/IIS/Centre Oscar Lambret/Lille University SMMiL-E Project, CNRS Délégation Hauts-de-France, 43 Avenue le Corbusier, 59800 Lille, France; [3]CNRS, IRL2820, Laboratory for Integrated Micro Mechatronic Systems, Institute of Industrial Science, University of Tokyo, 4-6-1 Komaba, Meguro-ku, Tokyo 153-8505, Japan; [4]University Lille, CNRS, INSERM, UMR8199-1283, EGID, Institut Pasteur de Lille, EGID, Lille University Hospital, 59000 Lille, France; [5]University Lille, INSERM, CHU Lille, U1192 - Protéomique Réponse Inflammatoire Spectrométrie de Masse - PRISM, 59000 Lille, France; [6]University Lille, CNRS, INSERM, CHU Lille, Institut Pasteur de Lille, US 41 - UAR 2014 - PLBS BICeL, 59000 Lille, France; [7]University Rennes, INSERM, EHESP, Irset (Institut de Recherche en Santé, Environnement et Travail) - UMR_S 1085, 35000 Rennes, France; [8]University Rennes, CNRS, INSERM, Biosit UAR 3480 US_S 018, Protim Core Facility, 35000 Rennes, France; [9]Aix Marseille Université, CNRS, INSERM, CIML, 13288 Marseille Cedex 9, France; [10]Institute of Biomedicine (iBiMED), Department of Medical Sciences, University of Aveiro, 3810-193 Aveiro, Portugal; [11]Department of Metabolism, Imperial College London, Hammersmith Hospital, London W12 ONN, UK; [12]Institut Pasteur de Lille – PLEHTA (Plateforme d'Expérimentation et de Haute Technologie Animale), 59019 Lille, France; [13]Shanghai Institute of Immunology, Department of Microbiology and Immunology, Shanghai Jiao Tong University School of Medicine, Shanghai 200025, P.R. China; [14]Institut Universitaire de France, Ministère de l'Enseignement Supérieur, de la Recherche et de l'Innovation, 1 Rue Descartes, 75231 Paris Cedex 5, France

Immuno-oncotherapy is a highly promising therapeutic strategy that relies on the ability of cancer cells to present specific antigenic epitopes at their surfaces. Because they proliferate rapidly, cancer cells frequently accumulate genetic variants, including premature termination codons. In this study, we investigated the potential of 2,6-diaminopurine (DAP), a potent translational-readthrough-inducing molecule, to elicit an antitumor immune response. Readthrough-resulting proteins following DAP treatment can be displayed at the cell surface by the major histocompatibility complex, thus potentially enhancing immune recognition. This was demonstrated using a construct encoding FIREFLY LUCIFERASE interrupted by a UGA stop codon and fused at its C terminus with the SL8 antigenic peptide. Furthermore, *in vivo* exposure to DAP promotes the recruitment of immune effector cells, including T lymphocytes, macrophages, and natural killer cells, to the tumor microenvironment. These findings suggest that DAP and potentially other translational readthrough-inducing molecules hold promise as novel candidate drugs for antitumor therapy.

INTRODUCTION

Despite significant advances in oncological diagnostics and therapies, cancer remains a leading cause of mortality. One of the most promising therapeutic strategies is immunotherapy, which aims to harness the immune system to mount an antitumor response. A key challenge of this approach is to enable tumor cells to generate specific, distinct antigen epitopes capable of triggering a targeted immune reaction.[1–3]

A distinguishing feature of tumor cells is their elevated rate of cell division as compared to healthy cells. This rapid proliferation results in the accumulation of approximately 100 new genomic variants with each division. While many of these variants occur in non-coding intergenic regions, they can affect coding regions as well. Such variants can display insertions, deletions, splicing defects, or point variants, among others. These genetic alterations often lead to frameshifts, resulting in the introduction of premature termination codons (PTCs).

The presence of a PTC in mRNA activates a surveillance mechanism known as nonsense-mediated mRNA decay (NMD).[4–7] NMD specifically identifies and degrades PTC-containing mRNAs, effectively silencing these transcripts to protect cells from potential harmful effects caused by the synthesis of truncated proteins. However,

Received 27 March 2025; accepted 10 September 2025;
https://doi.org/10.1016/j.ymthe.2025.09.024.

Correspondence: Fabrice Lejeune, University Lille, CNRS, INSERM, UMR9020-U1277 - CANTHER - Cancer Heterogeneity Plasticity and Resistance to Therapies, 59000 Lille, France.
E-mail: fabrice.lejeune@inserm.fr

 © 2025 The Authors.
Published by Elsevier Inc. on behalf of The American Society of Gene and Cell Therapy.
This is an open access article under the CC BY license (http://creativecommons.org/licenses/by/4.0/).

inducing the synthesis and translation of PTC-containing mRNAs could offer a novel approach to synthesizing chimeric proteins with unique C-terminal regions present only in tumor cells with a high mutational burden and thus not in healthy cells.[8] When epitopes derived from these tumor-specific C-terminal regions are processed and presented by major histocompatibility complex class I (MHC class I) molecules, they are recognized as non-self-peptides and elicit a cytotoxic T cell response against tumor cells producing PTC-containing mRNAs. This concept was proposed and demonstrated by Gilboa's team, who showed that targeting SMG1 or UPF2—two central components of the NMD pathway—using RNA interference can activate a $CD8^+$ T lymphocyte-dependent immune response.[9] A recent study in which NMD inhibition by 5-azacytidine was used to generate neoantigens on the tumor-cell surface has confirmed the validity of this approach.[10]

Translational readthrough is a mechanism allowing the ribosome to recruit a tRNA charged with an amino acid when it pauses at a PTC.[11,12] Activating readthrough can result in the synthesis of proteins with extended C-terminal regions, whose efficacy might potentially surpass that of NMD inhibitors. This approach is particularly promising because generating longer aberrant peptides increases the likelihood of producing antigenic peptides capable of eliciting an antitumor immune response.

In the present study, we tested this strategy using 2,6-diaminopurine (DAP) to promote translational readthrough for effectively inducing a tumor cell-targeting immune response. DAP, a recently identified molecule, has demonstrated efficacy both *in vitro* and *in vivo*[13–15] and appears to be more potent than other readthrough-inducing compounds.[16] Importantly, we found that DAP did not alter blood composition or impair vascular integrity. Instead, it promoted recruitment of various immune cell types to the tumor microenvironment and appears as a promising drug candidate for eliciting a specific antitumor immune response.

RESULTS

Cancer cells accumulate PTCs, whereas DAP treatment causes no increase in PTC number

The principle of neoantigen synthesis through translational readthrough relies on greater accumulation of PTCs in tumor cells than in healthy cells, due to the former cells' higher division rate. It was therefore critical to evaluate the disparity between tumor and non-tumor cells in PTCs introduced by insertion or deletion or splicing default. For this, 1 million CT26 colon carcinoma cells were subcutaneously injected into syngeneic Balb/C mice to induce tumor development at the injection site. Starting on the day after cell injection, the mice were treated daily by oral gavage with either a DMSO solution (control mice, $n = 9$) or a DAP solution ($n = 8$). Tumors and healthy lung tissue samples were collected 16 days after initial cell injection, and their DNA was extracted. Genetic variants were identified by whole-exome sequencing in all samples (Figure 1). The number of somatic PTCs present in tumor cells but not in healthy cells was compared with the number of somatic PTCs found in healthy cells but not in tumor cells. In control mice, comparative analysis revealed 2.6 times as many "tumor-only" somatic PTCs (mean: 157 ± 10) as "healthy cell-only" somatic PTCs (mean: 59 ± 5) (Figure 1A). This confirms that tumor cells accumulate many more somatic variants, including PTCs, than healthy cells. To test the mutagenicity of DAP in our model, a similar comparative PTC analysis was performed on tumor samples and healthy tissue from DAP-treated versus DMSO-treated mice. As shown in Figure 1B, we observed no difference in somatic PTC counts between DAP-treated and DMSO-treated mice in either tumor cells (mean: 164 ± 7 versus 157 ± 10) or healthy lung cells (61 ± 5 versus 59 ± 5). We conclude that DAP is not mutagenic in our model, in keeping with previous results.[14]

DAP has a limited impact on overall protein levels

Before using DAP to induce neoantigen synthesis, it was essential to confirm that this molecule did not significantly disrupt overall protein homeostasis. To address this, mass spectrometry (MS) was used to compare the proteomes of DAP-treated and untreated cells. The study was performed on HeLa and HDQ-P1, two widely used cancer cell lines, at three concentrations surrounding the effective dose of DAP.[14] The number of proteins showing altered levels in response to DAP was found to increase with the molecule's concentration (Figure 2; Tables 1 and S1). Specifically, the number of affected proteins ranged from a few dozen at 5 μM DAP to somewhat fewer than 250 at 500 μM DAP among the almost 6,800 proteins quantified. This suggests that less than 4% of the analyzed proteome was impacted at 500 μM DAP, assuming one protein per gene. Notably, the number of affected proteins was 2–4 times as high in HeLa cells as in HDQ-P1 cells. This difference may be attributable to a higher mutation burden in HeLa cells, which were established in 1951, as compared to the more recently established HDQ-P1 cells.[17] Further analysis revealed that very few proteins were affected across all 3 DAP concentrations tested: 11 proteins in HeLa cells and 3 in HDQ-P1 cells. Only a small subset of affected proteins was shared between the two cell lines (Figure 2). These findings suggest that DAP has minimal impact on the proteome and does not cause widespread changes in protein levels (Figure S2).

DAP does not modify the composition of blood

To ensure that the presence of DAP in an organism does not inherently disrupt the immune system, causing over- or underrepresentation of certain immune cell types, it was deemed necessary to study the immune cell composition of peripheral blood in Balb/C mice treated daily for 6 months by gavage with a DMSO solution or with 1 mg DAP (Figures 3A and 3B). Mouse weight was monitored weekly, and blood analyses were done monthly. The results indicated that DAP treatment did not significantly disrupt either the weight curve (Figure 3A) or blood composition. Red and white blood cell counts and numbers of monocytes, lymphocytes, eosinophils, basophils, and neutrophils (Figure 3B) were found to be similar in control and DAP-treated mice.

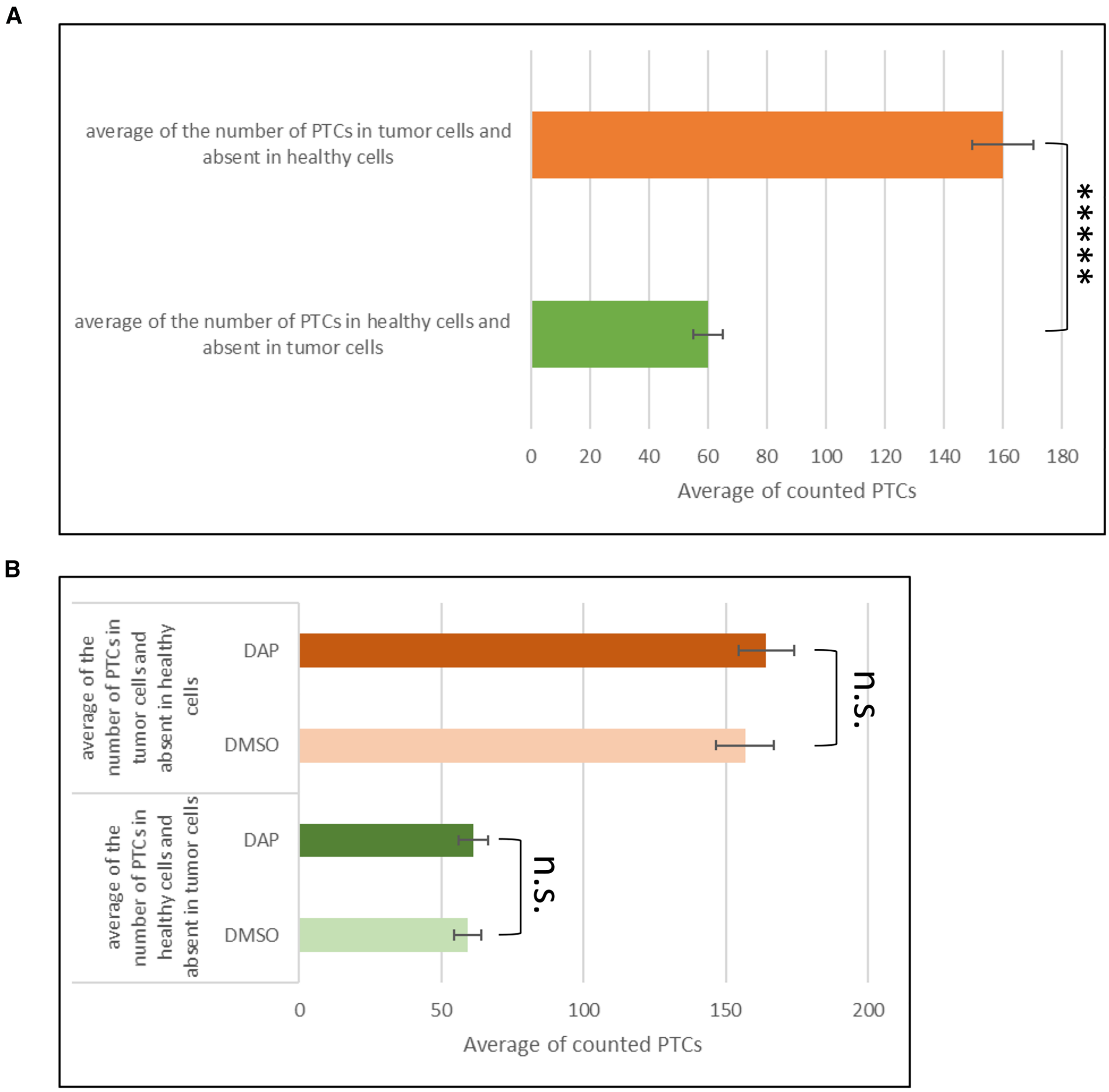

Figure 1. PTCs are more frequent in cancer cells than in healthy cells

Balb/C mice were injected with CT26 cells before being treated with DMSO or DAP on a daily basis. The number of PTCs was detected by next-generation sequencing in the CT26 tumor cells and in lung cells considered to be healthy cells. (A) Bar plot comparing the number of PTCs found in healthy cells but not tumor cells (17 mice for each cell group). (B) Analysis of the number of PTCs found in mice treated with DMSO (nine mice) or DAP (eight mice). p values were calculated by t test, *****$p < 0.0001$; n.s., non-significant.

DAP does not impair blood vessel integrity

The recruitment of immune cells into a tumor depends on the integrity of the vascular barrier, especially that of inter-endothelial cell junctions, which are crucial to immune cell diapedesis.[18] To ensure that DAP does not alter the integrity of blood vessels, we used primary human endothelial cells to construct three-dimensional blood vessels-on-chip with a functional vascular barrier.[19] Established vessels were then treated with drugs for 24 h and tested for permeability by monitoring the rate of diffusion of dextran-Texas red, 70 kDa out of the vessels, as dextran leakage from the inside to the outside of the vessel indicates that the drug disrupts the structure of the vessel wall by affecting cell junctions. The presence of DAP or G418, another readthrough-inducing compound, was found not to alter significantly either the rate of dextran diffusion nor the calculated diffusion permeability coefficient. This indicated that neither of these molecules affected the integrity of the vascular barrier, in contrast to thrombin used as a positive permeability agonist, which is known to impact the integrity of the blood vessel wall (Figure 3C).

DAP enables antigen presentation at the cell surface following translational readthrough activation

Previous studies have demonstrated that NMD inhibition can lead to the presentation of peptides at the cell surface by MHC class I.[9,20] Translational readthrough activation could also favor the

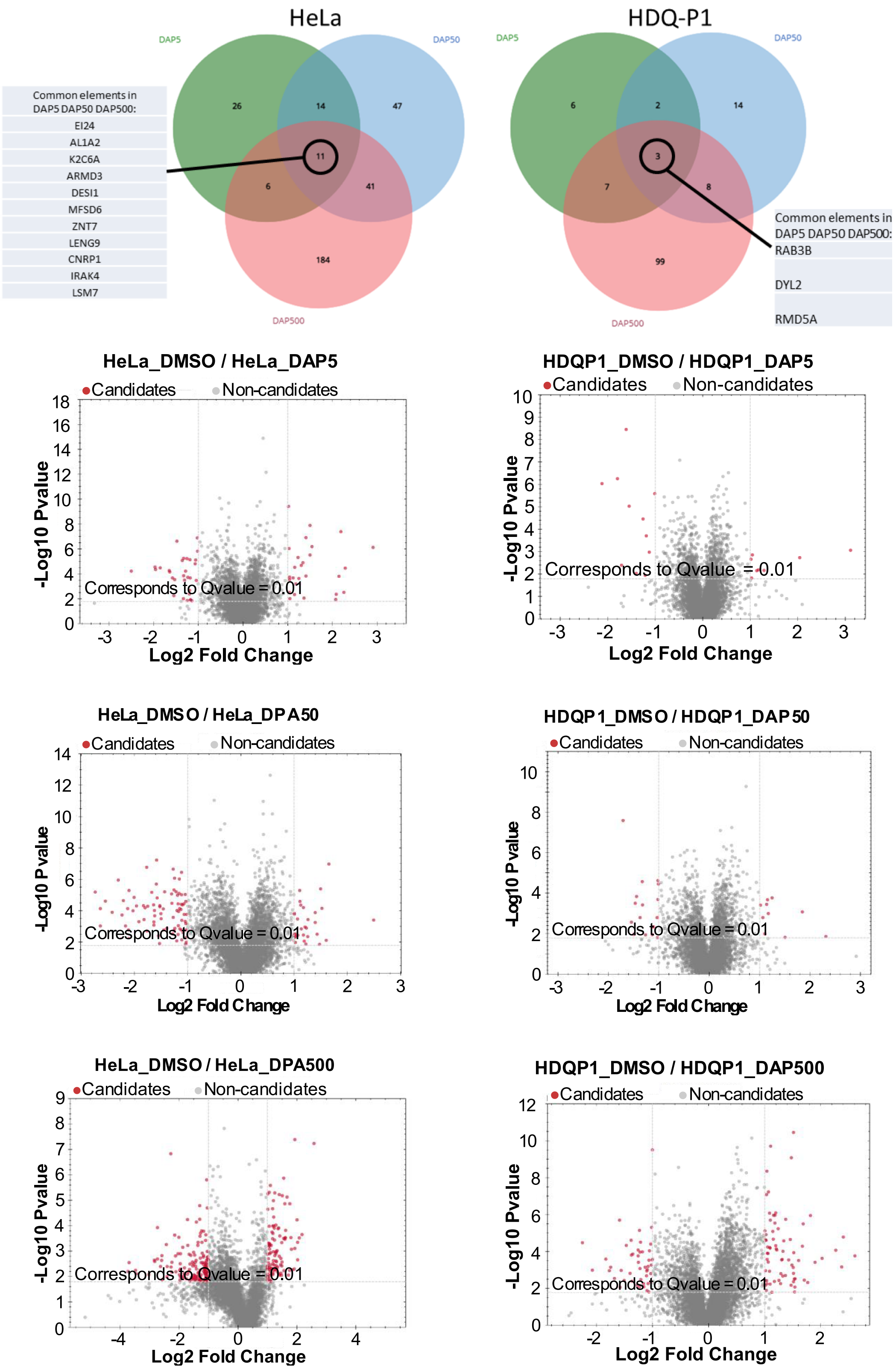

(legend on next page)

presentation of cryptic antigenic peptides by MHC class I,[21] and DAP could enhance strongly the presentation of new antigenic epitopes in cancer cells presenting a high mutation burden. To investigate this, a previously described construct[22] encoding firefly luciferase interrupted by a premature UGA stop codon was modified in the C-terminal region to express the antigenic peptide SL8 of OVALBUMIN (Ova 257–264, SIINFEKL), which can be easily tracked by cytometry upon presentation by mouse MHC class I (Figure 4A).

U2OS human osteosarcoma cells were transfected with this construct along with an expression vector coding for the murine MHC protein H2Kb. The cells were then treated for 24 h with DMSO, DAP, or G418, and luciferase activity was determined as a measure of readthrough efficiency (Figure 4B). As previously reported, the recorded firefly luciferase activity was higher in the presence of DAP than in the presence of G418 and higher in the presence of G418 than after DMSO treatment.[14] Flow cytometry analysis with the SL8-K^b complex-specific antibody 25D1.16[23] was then performed to detect the presence of SL8 presented by H2Kb at the surfaces of U2OS cells (Figure 4C). The results show that DAP treatment induced SL8 peptide processing and presentation by MHC class I more effectively than did G418, in correlation with the intensity of readthrough induced by the two drugs (Figure 4B). These results demonstrate that DAP effectively promotes the synthesis of neo-proteins through translational readthrough and facilitates MHC class I-restricted presentation of associated antigenic peptides.

DAP causes a delay in tumor growth in immunocompetent mice but not in immunodeficient mice

By inducing translational readthrough, DAP treatment leads to the presentation of neoantigens at the cell surface (Figure 4). These neoantigens are likely to trigger an antitumor immune response, expected to affect tumor growth. To test this, CT26 murine colon carcinoma cells syngeneic to Balb/C mice were injected into immunodeficient NGS mice and immunocompetent Balb/C mice. After injection, the mice were gavaged daily either with DMSO solution or with 1 mg DAP. Tumor growth was then monitored over a 2-week period (Figure 5). In immunodeficient mice, tumor growth appeared the same regardless of treatment. In immunocompetent mice, in contrast, a significant slowdown of tumor growth was observed with DAP treatment. These results indicate that DAP can exert an antitumor effect in the presence of a functional immune system.

Characterization of the antitumor immune response induced by DAP

The antitumor effect of DAP observed in immunocompetent Balb/C mice (Figure 5) was further characterized by studying the presence of different immune cells within the tumor, with and without DAP treatment. CT26/Balb/C tumors were collected and analyzed by immunohistochemistry to detect the presence of lymphocytes, macrophages, and natural killer (NK) cells. The following markers were used: CD4 or CD8 for T lymphocytes, CD19 for B lymphocytes, NKp46 for NK cells, and F4/80 for macrophages (Figure 6).

Table 1. Number of proteins whose expression was modified upon DAP treatment versus DMSO treatment

	HeLa	HDQ_P1
DMSO/DAP 5	57	17
DMSO/DAP 50	113	26
DMSO/DAP 500	242	116

A comprehensive analysis of tumor samples from DAP-treated versus control mice revealed a significant increase in $CD4^+$ and $CD8^+$ T lymphocytes, NK cells, and macrophages. The number of B lymphocytes did not appear to increase following DAP treatment. The recruitment of diverse immune cell types to the tumor in DAP-treated mice supports the hypothesis that DAP can trigger activation of an antitumor immune response. The observed immune cell types are consistent with those described as being involved in the antitumor immune response.[24] Interestingly, analysis of a healthy tissue, the lungs, revealed no increase in $CD8^+$ lymphocytes, NK cells, or macrophages following DAP treatment, thereby excluding a generalized increase in immune cell numbers across all tissues (Figure S1).

Furthermore, analysis of INTERFERON-γ (IFN-γ) and INTERLEUKIN-10 (IL-10) synthesis demonstrated an increase in IFN-γ but not IL-10 following DAP treatment, suggesting that the immune cells recruited to the tumor are activated and exert an antitumor effect (Figure S2).[25,26] This induction of IFN-γ but not IL-10 in the presence of DAP can be recapitulated by co-culturing CT26 cells with white blood cells isolated from the spleens of untreated Balb/C mice (Figure S3).

The antitumor immune response induced by DAP is linked to recognition of tumor cells by the immune system

To rule out the possibility that DAP might activate the immune system independently of neoantigen production, the following experiment was carried out. First, immunocompetent Balb/C mice received, in the right flank, a subcutaneous injection of CT26 cells that had been treated for 24 h with either DAP or DMSO. Eight days after this injection, each mouse received a second subcutaneous injection, this time in the left flank, of either DAP-treated or DMSO-treated CT26 cells. Four study groups were thus formed: mice having received DMSO-treated CT26 cells in both injections (DMSO/DMSO group), mice having received DMSO-treated cells

Figure 2. DAP has a limited impact on the proteome

The Venn diagrams show the number of proteins whose levels are modified in DAP-treated cells (at 5 μM (DAP 5), 50 μM (DAP 50), or 500 μM (DAP 500) versus DMSO-treated cells. A table is shown for each cell type, with the identities of proteins showing altered expression at all three DAP concentrations. At bottom are volcano plots of altered protein expression for each DAP concentration. The red dots indicate proteins showing a significant difference in expression versus DMSO-treated cells.

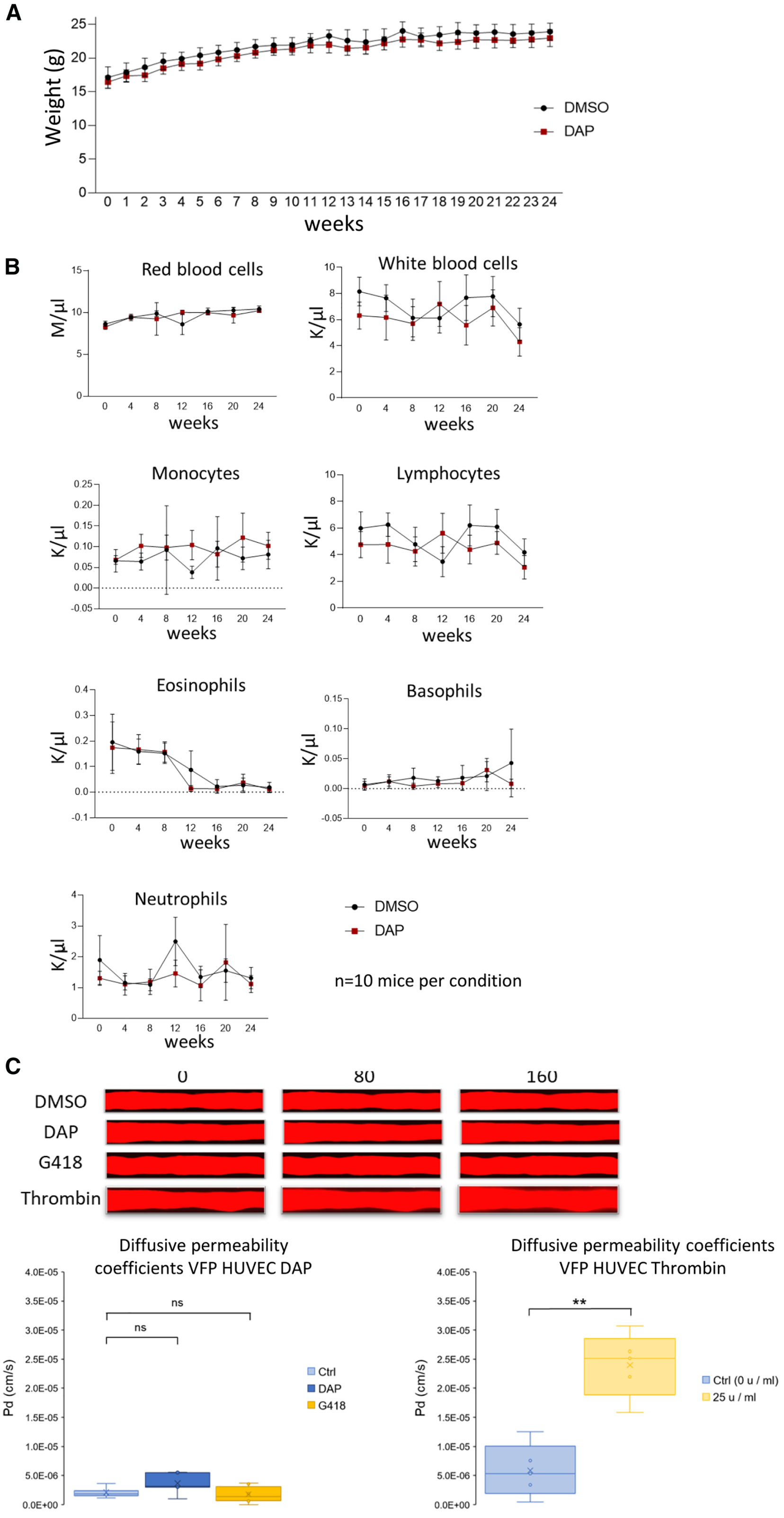

Figure 3. DAP does not alter blood composition

(A) Weight curves of mice treated daily with DMSO (black circles) or DAP (red squares) for 6 months. Each mouse was weighed weekly. (B) Blood composition of mice treated with DAP (red squares) or untreated (black circles). (C) Top: blood vessels-on-chip were treated with drugs for 24 h before injection of dextran 70 kDa Texas red into the lumens. Video recording of dye diffusion was performed over 160 s under lighting appropriate for fluorescence emission. Images illustrate blood vessels at injection time (0) and at 80 s and 160 s post-injection. Bottom: diffusive permeability coefficients calculated from the diffusion curves for each treatment (n = 4–6). p values were calculated by t test, n.s., non-significant.

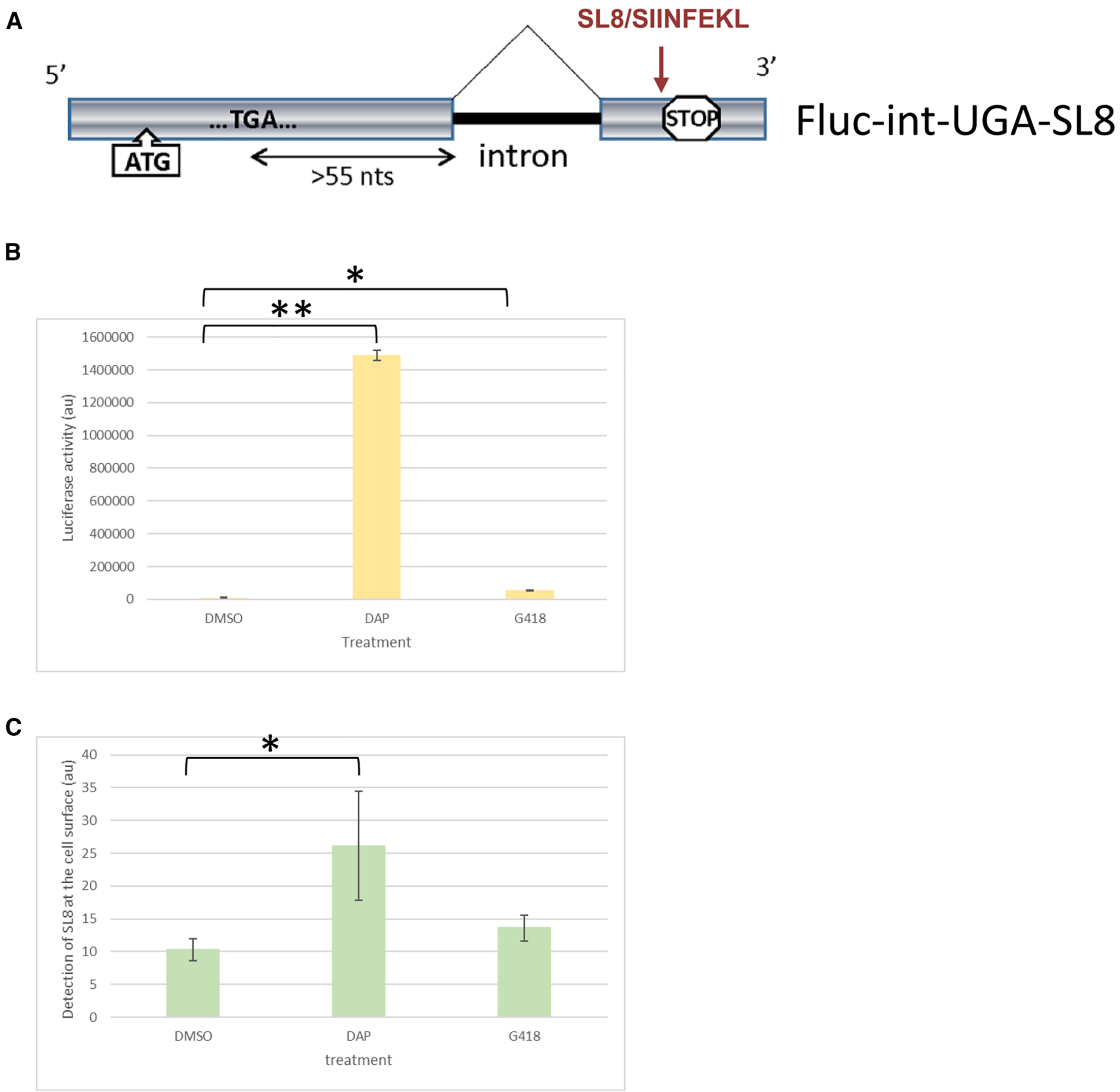

Figure 4. The C-terminal region of a protein produced through readthrough can be presented at the cell surface by MHC class I

U2OS cells were transfected with the expression vector described in (A) along with a plasmid encoding the murine MHC class I molecule H-2KB. Following transfection, cells were either subjected to a 24-h treatment with DMSO, DAP, or G418 to assess translational readthrough efficiency or analyzed for the presence of the SL8 peptide presented on surface-expressed H2-κb molecules. (A) Schema of the construct used to express an mRNA encoding FIREFLY LUCIFERASE with a PTC followed by the SL8 peptide sequence. (B) Readthrough efficiency determined by measuring luciferase activity in U2OS cells transfected with the construct described in (A) in the presence of DMSO, 100 μM DAP, or 600 μM G418. (C) Flow cytometry analysis of SL8 presence at the surfaces of cells transfected with the construct described in (A) and incubated with DMSO, 100 μM DAP, or 600 μM G418. *p* values were calculated by *t* test, *$p < 0.05$, **$p < 0.01$.

first and DAP-treated cells later (DMSO/DAP group), mice having received DAP-treated cells first and DMSO-treated cells later (DAP/DMSO group), and mice having received DAP-treated cells in both injections (DAP/DAP group). The mice did not receive any further treatment. Tumors were collected 15 days after the second injection for immunohistochemical analysis (Figure 7A).

Examination of the tumors derived from the first injection (Figure 7B) revealed the following: (1) $CD8^+$ T lymphocytes were recruited to the tumor in all instances where DAP-treated cells had been injected, whether in the first injection, the second, or both; (2) the presence of macrophages (F4/80 marker) was observed only when the cells of the first injection had been treated with DAP; (3) the presence of NK cells (NKp46 marker) was observed when the second injection contained DAP-treated cells; and (4) no increase in $CD4^+$ T lymphocytes or B lymphocytes (CD19 marker) was observed in these tumors.

Examination of the tumors derived from the second injection (Figure 7C) revealed the following: (1) significant recruitment of

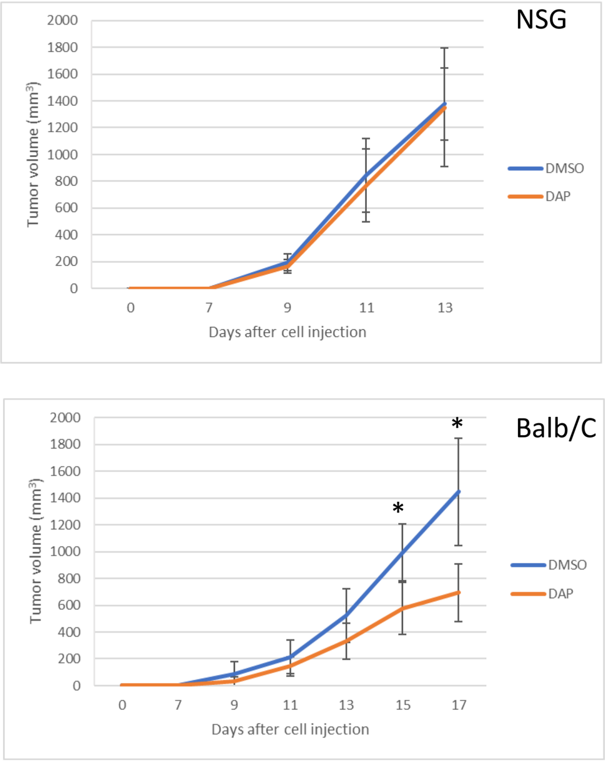

Figure 5. DAP affects tumor growth in immunocompetent but not in immunodeficient mice

Immunodeficient NGS mice (top; nine mice per treatment) or immunocompetent Balb/C mice (bottom; seven mice per treatment) were subcutaneously injected with 10 million CT26 cells. Tumor growth was measured until tumor size reached the endpoint in one of the treatment groups (13 days after cell injection for NGS mice, 17 days for Balb/C mice). The t test was used to calculate $*p < 0.05$.

$CD8^+$ T lymphocytes, NK cells, and macrophages whenever DAP-pretreated cells were injected, whether in the first injection, the second, or both; (2) $CD4^+$ T lymphocytes were detected when the second injection contained DAP-pretreated cells; and (3) no increase in B lymphocytes was observed.

Interestingly, increased IFN-γ expression was observed in both the first and second tumors in all mice having received at least one injection of DAP-treated cells. This increase was most pronounced when DAP-treated cells were injected twice (Figure S4). In contrast, neither the first nor the second tumors showed any increase in INTERLEUKIN-10 expression when DAP-treated cells were injected, regardless of whether this occurred at the first injection, the second, or both. On the contrary, by comparison with the situation where both tumors derived from DMSO-treated cells, the trend was toward a decrease. This is consistent with activation of an immune response (Figure S5). These results indicate that the immune cells recruited to the tumor are activated and support the antitumor effect observed in Figure 5.

Taken together, the results of Figures 7, S4, and S5 show that DAP-induced activation of an antitumor immune response is mediated by tumor cells. They indicate that DAP acts on these cells and not directly on the immune system. In addition, these results confirm that immune cell recruitment exerts an antitumor effect based on increased synthesis of IFN-γ and decreased synthesis of IL-10 at the tumor site.

DISCUSSION

Developing an immunotherapeutic approach to treat cancer requires expression of neoantigens at the surface of cancer cells, triggering an immune response. Like NMD inhibition, translational readthrough activation can promote cell-surface neoantigen expression. In theory, it should be more effective than NMD inhibition, because it results in the production of an aberrant C-terminal region extending beyond the PTC, longer than that produced by NMD inhibition.

In this study, we have used whole-exome sequencing to show that cancer cells accumulate PTCs (Figure 1). It is worth noting, however, that PTCs were also found, although at lower levels, in healthy tissue. This may be due to our use of a syngeneic model with tumor cells whose genome is not identical to that of the host organism. The results in Figure 3 show that these PTCs in healthy cells do not trigger immune system activation upon DAP treatment, ruling out a possible autoimmune reaction induced by the expression of neoantigens at the surface of healthy cells.[21] This may be due to the fact that these mutations have been present since early development, and as baseline readthrough occurs in all cells,[11,12] these mutations do not generate non-self-peptides.

Prior to establishing that DAP can induce an antitumor immune response, several validations were necessary. Among these, we showed that the impact of DAP on the proteome is minimal, with less than 4% of analyzed proteins showing altered levels in its presence (Figure 2; Tables 1 and S1). One might have expected the appearance of proteins with aberrant C-terminal regions due to the introduction of PTCs in certain mRNAs. However, such peptide sequences are not found in databases compiling peptides from wild-type protein sequences. Therefore, the analysis presented in Figure 2 was solely intended to assess the impact of DAP treatment on overall proteome expression. Importantly, DAP did not affect levels of immune-cell-related proteins. We have also obtained essential confirmation that DAP itself did not alter the cell composition of blood, even after 6 months of exposure to this readthrough-inducing molecule (Figure 3).

For DAP to induce an antitumor immune response, it must facilitate the presentation of peptides encoded downstream of a PTC. Our results clearly demonstrate that under DAP treatment, translational readthrough of a construct encoding firefly luciferase interrupted by a PTC and ending with the SL8 ovalbumin-derived peptide results in efficient presentation of the SL8 peptide at the cell surface. This ability of DAP to promote neoantigen presentation on the cell surface through translational readthrough, combined with the accumulation of PTCs in tumor cells, accounts for the significant delay in tumor growth observed in DAP-treated immunocompetent mice

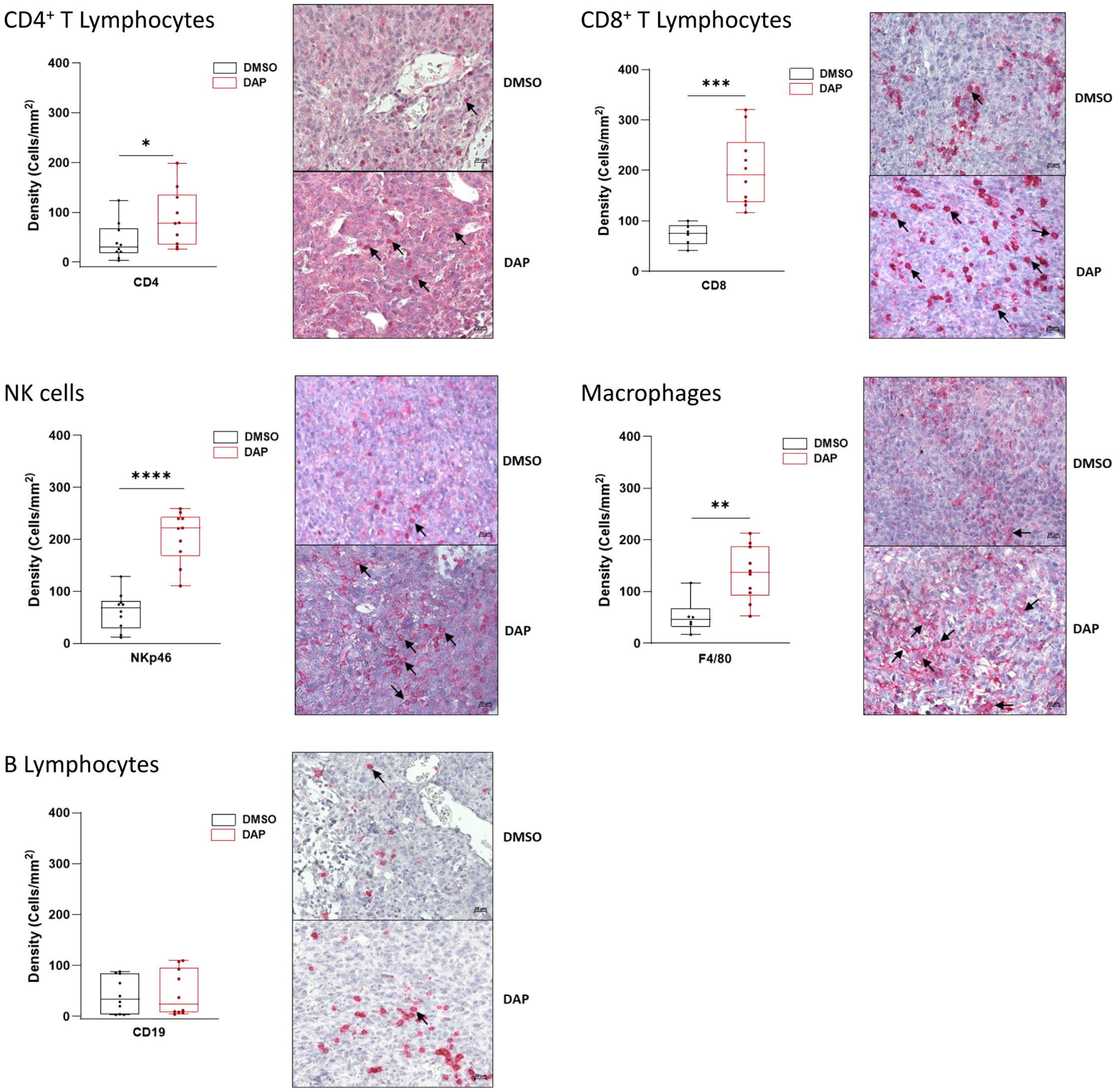

Figure 6. DAP induces immune cell recruitment to the tumor

Immunocompetent Balb/C mice were injected with 10 million CT26 cells and then treated orally each day with either DMSO (negative control) or 1 mg DAP (nine mice per group). Seventeen days after injection, tumors were collected for immunohistochemistry to detect the presence of CD4+ T lymphocytes (top left, CD4), CD8+ T lymphocytes (top right, CD8), natural killer cells (center left, NKp46), macrophages (center right, F4/80), or B lymphocytes (bottom, CD19). Arrows indicate examples of positive cells. Quantifications are shown to the left of each image. p values were calculated by t test, $^{*}p < 0.05$, $^{**}p < 0.01$, $^{***}p < 0.001$, $^{****}p < 0.0001$.

(Figure 5) and the recruitment of immune cells to the tumor site (Figure 6) but not in healthy tissue, such as lung for example (Figure S1). The nature of the immune cells recruited to the tumor under DAP treatment suggests the involvement of macrophages, NK cells, and $CD8^{+}$ T lymphocytes in the antitumor immune response.[9,24]

At the tumor site, recruited immune cells interact with the tumor cells, initiating a complex and dynamic immune response. The plasticity of immune cells and their interactions with all components within the tumor microenvironment are crucial in determining a trend toward an immunosuppressive or inflammatory environment. These dynamics ultimately affect the outcome, facilitating tumor clearance or contributing to tumor progression.[27,28] The lower level of IL-10 in the DAP-treated tumors or cells, combined with the rise in IFN-γ, suggests a more activated antitumor immune response (Figures S2 and S3). Furthermore, the increment in this important pro-inflammatory cytokine supports the idea that DAP prepares tumor cells for immune recognition presumably by improving the translation readthrough of PTCs and promoting the expression of neoantigens.

The antitumor immune response observed with DAP treatment is not due to the mere presence of the molecule in the organism but

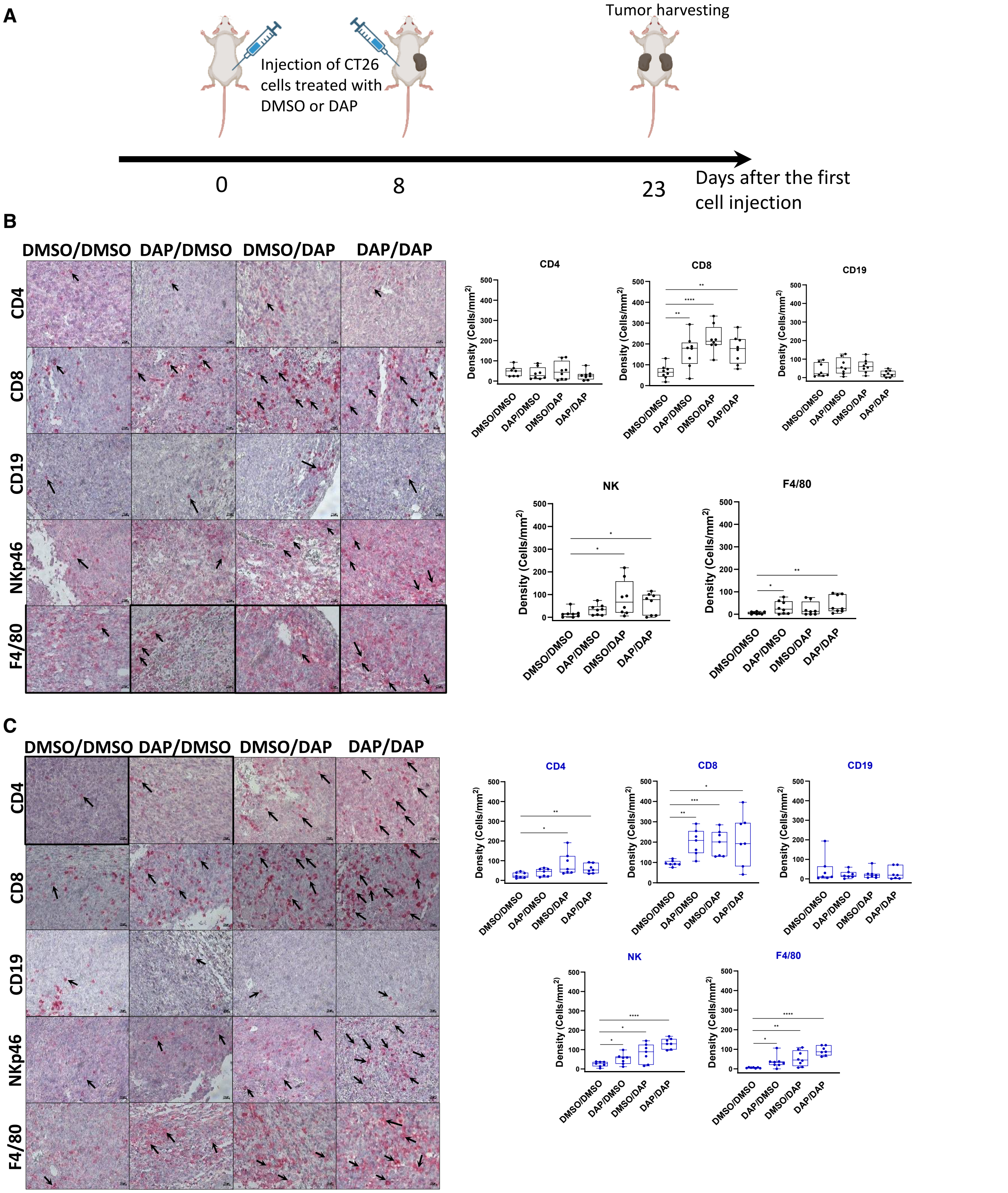

Figure 7. DAP-treated CT26 cells can recruit immune cells to the tumor site

Balb/C mice (nine per group) were injected with 10 million CT26 cells for each injection. (A) Schematic representation of the experimental procedure. (B) Immunohistochemistry performed on the tumor derived from the first injection of cells (first tumor) to detect the presence of $CD4^+$ T lymphocytes (top left, CD4), $CD8^+$ T lymphocytes (top right, CD8), NK cells (center left, NKp46), macrophages (center right, F4/80), or B lymphocytes (bottom, CD19). Arrows indicate examples of positive cells. Quantifications are shown to the left of each image. (C) Analysis similar to that shown in (B), but performed on the tumor derived from the second injection of CT26 cells (second tumor). *p* values were calculated by *t* test, $*p < 0.05$, $**p < 0.01$, $***p < 0.001$, $****p < 0.0001$.

likely to its readthrough activity, leading to the presentation of neoantigens on tumor cell surfaces. This is apparent from what happens when immunocompetent mice are not DAP treated but are injected with cancer cells pretreated with DAP so as to favor neoantigen synthesis; an immune response is activated (Figure 7B), similar in nature to the response seen in DAP-treated mice (Figure 6). Interestingly, when a second injection of cancer cells is administered 1 week after the first injection, an even greater recruitment of $CD8^+$ T lymphocytes to the tumor is observed (Figure 7C). As previously described in the literature, $CD8^+$ T cells, known for their cytotoxic functions, are key players in the immune response against cancer. They produce cytotoxic molecules such as granzyme B, perforin, and IFN-γ, which act in concert to eliminate tumor cells.[29–32] It is noteworthy that in our experiments on mice, when a tumor was generated from one injection of DAP-treated cells (either the first or the second) and the other injection contained DMSO-treated cells, immune cells were still recruited to the tumorigenesis site. This indicates that these immune cells were indeed activated to target tumor cells. This active status of the immune cells was reinforced by synthesis of IFN-γ and a decrease in IL-10 (Figures S4 and S5).

IFN-γ plays a critical role in amplifying the cytotoxic effector functions of NK cells and $CD8^+$ T lymphocytes, two crucial effectors of antitumor immunity.[33] Furthermore, IFN-γ significantly enhances the immunogenicity of tumor cells by upregulating the expression of MHC class I[34–36] and thereby making the cells more visible to the immune system and susceptible to destruction.[37–40]

The present demonstration of the potential to exploit DAP in immuno-oncology likely on the basis of its ability to activate translational readthrough was probably made possible by the high readthrough efficiency of DAP.[14,16] It is uncertain whether molecules with significantly lower readthrough efficiencies would have a similar effect. However, DAP induces readthrough only at UGA PTCs. This immuno-oncological approach could potentially be optimized by using a readthrough-inducing molecule having an efficiency similar to that of DAP but targeting all three types of PTCs (UGA, UAG, and UAA). Such a molecule remains to be identified and could help validate our hypothesis that the activation of translational readthrough triggers an immune response against tumor cells by inducing the synthesis of neoantigens. The use of the syngeneic CT26/Balb/C syngenic model was motivated by previous reports indicating that NMD inhibition can elicit an antitumor immune response, likely through the induction of neoantigen expression.[9] Notably, this neoantigen-driven antitumor response following NMD inhibition has been shown to be even more robust in the MC38/C57BL/6 syngeneic model, as recently reported.[41] This difference may be explained by the fact that CT26 cells are microsatellite stable, whereas MC38 cells are microsatellite unstable (MSI), a feature associated with higher tumor mutational burden and enhanced immunogenicity. Therefore, it cannot be excluded that our findings would be even more pronounced in an MSI-context tumor model. Nevertheless, the present study highlights the potential of readthrough-inducing molecules in cancer therapy, as previously shown for the treatment of other genetic diseases such as cystic fibrosis.[13,15]

MATERIALS AND METHODS

Whole-exome sequencing of tumors and healthy lung samples from Balb/C mice treated or not with DAP

One million CT26 colon carcinoma cells were subcutaneously injected into Balb/C mice (a syngeneic model) to induce tumor development at the injection site. Starting on the day after cell injection, the mice were treated daily by oral gavage with either 200 μL of a solution containing 10% dimethyl sulfoxide/90% phosphate-buffered saline as a negative control (n = 9) or 200 μL of a 5-mg/mL DAP solution (n = 8). A tumor sample and a healthy lung sample were collected from each mouse 16 days after the initial cell injection for DNA extraction. Whole-exome sequencing was then performed with the Mouse Exome Panel (Twist), which covers 37.7 Mb of the mouse coding genome, in accordance with recommended guidelines. The libraries were sequenced on the NovaSeq 6000Dx system (Illumina) in paired-end mode with a 150-bp read length. Sequencing yielded an average of 37,508 Mb per sample (ranging from a minimum of 30,267 Mb to a maximum of 44,183 Mb). Additionally, the percentage of bases with a quality score ≥Q30 was above 93% across all samples, ensuring high-quality data. For detecting somatic PTCs, we used Mutect2 version 2.2 to perform a tumor/healthy tissue comparative analysis for each sample pair, followed by variant annotation with Ensembl VEP 106. The analysis focused on stop-gain variants.

Proteomic analysis

Proteomic analysis was performed at the PROTIM Core Facility in Rennes, France. Samples were placed in 30 mM Tris buffer pH 7.4 containing 8 M urea, 4% CHAPS, and protease inhibitors (1 mM EDTA, 0. 5 mM dithiothreitol [DTT], 1 mM 4-(2-aminoethyl) benzenesulfonyl fluoride hydrochlorideF), and 10 μM L-*trans*-epoxysuccinyl-leucylamido(4-guanidino)butane [E64]) and lysed on ice with an ultrasound processor (Bioblock Scientific) set at 6 pulses of 10 s at 40% amplitude. After centrifugation (15,000 × g for 15 min at 4°C) to remove cell debris and ultracentrifugation at 105,000 × g for 1 h at 4°C, the total protein concentration of each sample was determined with a bicinchoninic acid assay (Thermo Fisher Scientific) according to the manufacturer's instructions. Cytosoluble proteins were stored at −80°C until proteomic analysis. Ten micrograms of each protein lysate underwent enzymatic digestion as previously described,[42] with minor modifications. Briefly, protein reduction was carried out for 15 min at 37°C in the presence of 7.2 mM DTT in UTH-PMax buffer (6 M urea, 50 mM Tris-HCl, 0.01% ProteaseMAX [Promega]). Alkylation was then performed for 15 min at room temperature and in the absence of light with 13.5 mM iodoacetamide in UTH-PMax buffer (see above). The samples were digested at 37°C for 3 h with 0.4 μg of a trypsin/Lys-C mix (V507A, Promega). After addition of 50 mM Tris-HCl pH 8 and 0.01% ProteaseMAX to reach 4 ng/μL enzyme, the digestion was continued overnight. The resulting peptide mixtures were then cleaned of salts, contaminants, and detergents by means of Phoenix

cartridges (PreOmics GmbH), and the final concentration of peptides was measured with a NanoDrop spectophotometer.

Around 300 ng peptides was analyzed by nanoliquid chromatography (nano-LC) and tandem MS (MS/MS) in the diaPASEF mode. The peptides were separated on a 75 μm × 250 mm IonOpticks Aurora 3 C18 column (Ion Opticks). A reverse-phase buffer gradient (buffer A: 0.1% formic acid, 2% acetonitrile, 97.9% H_2O; buffer B: 0.1% formic acid, 99.9% acetonitrile) was run at 50°C and at a flow rate of 250 nL/min on a nanoElute UHPLC System (Bruker Daltonik GmbH) controlled by HyStar software (version 6.0.30.0, Bruker Daltonik GmbH). The LC run lasted for 80 min. A starting concentration of 2% buffer B was used, increasing to 13% over the first 42 min. Buffer B concentrations were then increased up to 20% at 65 min, 30% at 70 min, 85% at 75 min, and finally, 85% for 5 min to wash the column. The temperature of the ion transfer capillary was set at 180°C. Ions were accumulated for 100 ms, and mobility separation was achieved by ramping the entrance potential from −160 to −20 V within 114 ms. The acquisition of the MS and MS/MS mass spectra was done with average resolutions of 50,000 full width at half-maximum (mass range 100–1,700 *m/z*), respectively. LC-MS/MS data were acquired with a data-independent acquisition method that uses parallel accumulation-serial fragmentation (DIA-PASEF). Thermal ionization MS (TIMS), MS operation, and the DIA-PASEF window were controlled and synchronized using the control instrument software timsControl version 5.1 (Bruker Daltonik GmbH).

For the development of the dia-PASEF method, we used the dia-PASEF method editor, and we manually defined a method to perform data-independent isolation of data from several 26 *m/z*-wide precursor windows, also called segments, in a single TIMS separation (0.74 ms) for a total of 6 segments and 29 boxes. These scans perfectly cover the diagonal area of doubly charged peptides and partially triply charged peptides in the *m/z* and ion mobility output range. MS and MS/MS data were collected over the *m/z* range 354.1–1,200 and over the mobility range from 1/K0 = 0.75 to 1/K0 = 1.33 Vs cm^{-2}. During each data collection, each TIMS cycle was 0.74 s long and comprised 1 MS segment and 6 cycles of dia-PASEF MS/MS segments, comprising 4 or 5 boxes, to cover the 29 boxes defined in the acquisition method. The collision energy was increased linearly with mobility from 59 eV at 1/K0 = 1.6 Vs cm^{-2} to 20 eV at 1/K0 = 0.6 Vs cm^{-2}. Ion-mobility-resolved mass spectra, nested ion mobility versus *m/z* distributions, and fragment ion intensity sums were extracted from the raw data file with DataAnalysis 6.0 (Bruker Daltonik GmbH). The signal-to-noise ratio was increased by summing the individual TIMS scans. Mobility peak positions and half-peak widths were determined from the extracted ion mobilograms (±0.05 Da) by means of the peak detection algorithm implemented in the DataAnalysis software. Feature detection was also performed with DataAnalysis 6.0 software and stored at the raw data level (.d). DIA raw files were analyzed in Spectronaut software version 18 (Biognosys), using the integrated Pulsar search engine. The calibration search was dynamic and the MS1, MS2 correction factor was 1. The data were searched against the Uniprot *Homo sapiens* UP000005640 database (one protein per gene, 20,593 sequences, downloaded in June 2023), with trypsin/P as the protease with up to one missed cleavage. To account for post-translational modifications and chemical labeling settings, carbamidomethylation of cysteine residues was defined as a fixed modification and methionine oxidation, as a variable modification. An false discovery rate of less than 1% was ensured at the precursor, peptide, and protein levels. For quantification: (1) single-hit proteins, defined as proteins identified with only one precursor, were excluded; (2) local regression normalization was carried out for the whole dataset; (3) run-wise imputation was performed for the missing data; (4) only proteotypic peptides specific for a protein group were used for quantification, and (5) the mean of the precursor quantities was used to determine peptide quantity, and the sum of peptide quantities was used to determine protein quantity. Finally, a t test was applied, and data were filtered using a q value (Storey multiple testing corrected *p* value) of 0.01 and an absolute log2 ratio of 1, corresponding to a fold change of 1.5.

Immunohistochemistry study

The *in situ* cellular immune response was characterized by immunohistochemistry. Briefly, the tissue samples were deparaffinized and rehydrated, after which peroxidase activity was blocked with 3% hydrogen peroxide. Antigen recovery was performed in Tris-EDTA buffer at pH 9.0 in a boiling water bath for 30 min.

The samples were then incubated overnight at 4°C with the following primary antibodies: CD4 (monoclonal, ab237722, Abcam), CD8 (monoclonal, ab217344, Abcam), CD19 (monoclonal, ab245235, Abcam), NCR1 (monoclonal, ab233558, Abcam), and F4/80 (monoclonal, ab111101, Abcam), at dilutions 1:150, 1:500, 1:200, 1:500, and 1:100, respectively. Isotype controls were employed as negative controls.

For all markers, SIGMAFAST Fast Red TR/Naphthol AS-MX tablets (catalog no. F4648, Sigma-Aldrich) were used according to the manufacturer's instructions. This was followed by counterstaining with Mayer's hematoxylin. Finally, the slides were dehydrated with an ascending series of alcohol and mounted with an aqueous mounting medium (F/AMT030) and a glass coverslip. Cells stained in red were identified as positive.

Quantitative morphometric analysis

Ten sequential fields of each histological section (40× magnification) were photographed by an optical microscope coupled to a computer using ZEN 3.10 software (Zeiss). Red-immunostained cells were quantified with ImageJ software, the cell color pattern and morphology being assessed for each antibody. Determination of cell density (cells per square millimeter) was calculated for each marker as the ratio of the number of immunostained cells to the area of each photo.

Measure of blood vessel integrity

Blood vessels-on-chip were prepared as before[19] and cultured for 3 days to allow proper establishment of the vascular barrier. Vessels were treated with drugs for 24 h and then injected with

50 µL 0.1 mg/mL dextran 70 kDa-Texas red solution (Thermo Fisher Scientific) in EGM-2. Diffusion was video recorded under fluorescent light with a Keyence BZ-X710 microscope. Diffusive permeability coefficients (*Pd*) were calculated by measuring over time differences between the average fluorescence intensity of the gel and that of vessel regions.[19]

Cell culture and cell treatment

U2OS or CT26 cells were grown in Dulbecco's modified Eagle's medium with GlutaMAX (Gibco) containing 10% fetal bovine serum (Sigma-Aldrich) and 1% ZellShield (Minerva Biolabs). These cells were transfected with JetOptimus (polyplus transfection). DAP (Sigma-Aldrich) was dissolved at 100 mM in DMSO or at 5 mg/mL in 10% DMSO/90% PBS (mouse treatments). G418 (GIBCO) was dissolved at 100 mg/mL in water. Primary human umbilical vein endothelial cells were cultured in EGM-2 according to the manufacturer's instructions and used between passages 1 and 7. All cells were cultured in humidified 95% air/5% CO_2 incubators at 37°C.

Flow cytometric analysis

Staining was performed on 10^6 U2OS cells for 30 min at 4°C in 50 µL PBS-2% serum with phycoerythrin-conjugated H-2KB-specific antibody (catalog no. 569791, BD Pharmingen). The cells were resuspended thoroughly, and 250 µL of fixation/permeabilization solution was added per condition prior to incubation for 20 min at 4°C. The cells were then washed twice in 1× BD Perm/Wash buffer. Data were recorded with the SP6800 Spectral Cell Analyzer (Sony Biotechnologies) at 488 nm excitation and analyzed with FlowJo software.

In vivo experiments

All *in vivo* experiments were conducted on 5-week-old female mice purchased from Charles River Laboratories (Balb/C and NGS). The study groups consisted of up to 10 mice, with fewer individuals if some were removed because of signs of distress. For xenografts, 10 million CT26 cells were injected subcutaneously. Tumor measurement was performed with a caliper. The formula applied was width × length × length. All experiments on animals have been approved by the ethics committee CEEA 75 (protocol 2022032914309195).

DATA AND CODE AVAILABILITY

All data are available in the main text or the supplemental information.

ACKNOWLEDGMENTS

C.S.P. is supported by the Agence Nationale de la Recherche (PEPR Biotherapy – projet RNAvac France 2030). A.M.L. is supported by a Ph.D. grant from Région Hauts-de-France and University of Lille. F.L. is supported by funding from Inca, La Comité 59 de la Ligue Contre le Cancer, the Agence Nationale de la Recherche (PEPR Biotherapy – projet RNAvac France 2030), Vaincre la Mucoviscidose, and the Association Française contre les Myopathies. The Canther Laboratory is part of ONCOLille Institute. This work was supported by a grant from Contrat de Plan Etat-Région CPER Cancer 2015–2020, a grant from La Ligue Contre le Cancer and INSERM (PRISM U1192 INSERM), a grant from the French National Research Agency (ProFI project ANR-24-INBS-0015), and funding from the Contrat de Plan Etat-Région 2021–2027 of the Hauts-de-France region. This work was partially funded by ChemBioFrance (Call 2023 Human Proteome Project - One Protein, One Ligand, One Function). As defined with ChemBioFrance, all proteomics analyses were performed by the PROTIM Core facility (Biosit - UAR 3480 CNRS US18 INSERM) using a standardized experimental design. The Protim Core Facility was supported by structural grants from Infrastructures en Biologie, Santé et Agronomie (IBiSA), the Regional Council of Bretagne (through Biogenouest), the European Regional Development Fund, Rennes Métropole, and the University of Rennes (through Biosit). M.D., B.T., P.F., and A.B. were supported by the National Center for Precision Diabetic Medicine – PreciDIAB, which is jointly supported by the French National Agency for Research (grant no. ANR-18-IBHU-0001), by the European Union (FEDER) (grant no. 20001891/NP0025517), by the Hauts-de-France Regional Council, and by the European Metropolis of Lille (grant no. MEL 2019_ESR_11). A.B was also supported by the European Research Council (grant no. OπO 101043671). We thank the France Génomique Consortium (ANR-10-INBS-009), Inca (PLBIO21-147), Agence Nationale de la Recherche (grant nos. ANR-22-PEBI-0007 and ANR-24-INBS-0015), La Ligue Contre le Cancer, the European Metropolis of Lille (grant no. MEL 2019_ESR_11), and the European Research Council (grant no. OπO 101043671).

AUTHOR CONTRIBUTIONS

C.S.P., A.M.L., M.D., C.L., and F.L. conceived the study. The methodology was developed by C.S.P., A.M.L., M.D., C.L., N.J., E.C., B.G., D.B., F.S., and F.L. The investigation was conducted by C.S.P., A.M.L., M.D., C.L., T.C., J.C., B.D., B.T., N.J., T.C., F.S., and F. L. Funding acquisition was carried out by R.B., P.F., C.P., P.P., F.S., I.V.S., R.L., M.S., A. B., and F.L. The project administration was managed by F.L., and supervision was provided by F.S., R.B., P.P., A.B., and F.L. The original draft of the manuscript was written by C.S.P., P.P., A.B., and F.L. All authors participated in the review and editing of the manuscript.

DECLARATION OF INTERESTS

The authors declare no competing interests.

SUPPLEMENTAL INFORMATION

Supplemental information can be found online at https://doi.org/10.1016/j.ymthe.2025.09.024.

REFERENCES

1. Galassi, C., Chan, T.A., Vitale, I., and Galluzzi, L. (2024). The hallmarks of cancer immune evasion. Cancer Cell *42*, 1825–1863. https://doi.org/10.1016/j.ccell.2024.09.010.
2. Kawakami, Y., Ohta, S., Sayem, M.A., Tsukamoto, N., and Yaguchi, T. (2020). Immune-resistant mechanisms in cancer immunotherapy. Int. J. Clin. Oncol. *25*, 810–817. https://doi.org/10.1007/s10147-019-01611-x.
3. Kumagai, S., Itahashi, K., and Nishikawa, H. (2024). Regulatory T cell-mediated immunosuppression orchestrated by cancer: towards an immuno-genomic paradigm for precision medicine. Nat. Rev. Clin. Oncol. *21*, 337–353. https://doi.org/10.1038/s41571-024-00870-6.
4. Carrard, J., and Lejeune, F. (2023). Nonsense-mediated mRNA decay, a simplified view of a complex mechanism. BMB Rep. *56*, 625.
5. Patro, I., Sahoo, A., Nayak, B.R., Das, R., Majumder, S., and Panigrahi, G.K. (2024). Nonsense-Mediated mRNA Decay: Mechanistic Insights and Physiological Significance. Mol. Biotechnol. *66*, 3077–3091. https://doi.org/10.1007/s12033-023-00927-4.
6. Sun, L., Mailliot, J., and Schaffitzel, C. (2023). Nonsense-Mediated mRNA Decay Factor Functions in Human Health and Disease. Biomedicines *11*, 722. https://doi.org/10.3390/biomedicines11030722.
7. Benslimane, N., Loret, C., Chazelas, P., Favreau, F., Faye, P.A., Lejeune, F., and Lia, A.S. (2024). Readthrough Activators and Nonsense-Mediated mRNA Decay Inhibitor Molecules: Real Potential in Many Genetic Diseases Harboring Premature Termination Codons. Pharmaceuticals (Basel) *17*, 314. https://doi.org/10.3390/ph17030314.
8. Lejeune, F. (2016). Triple effect of nonsense-mediated mRNA decay inhibition on cancer. Single Cell Biol. *5*, 136. https://doi.org/10.4172/2168-9431.1000136.

9. Pastor, F., Kolonias, D., Giangrande, P.H., and Gilboa, E. (2010). Induction of tumour immunity by targeted inhibition of nonsense-mediated mRNA decay. Nature *465*, 227–230. https://doi.org/10.1038/nature08999.

10. Becker, J.P., Helm, D., Rettel, M., Stein, F., Hernandez-Sanchez, A., Urban, K., Gebert, J., Kloor, M., Neu-Yilik, G., von Knebel Doeberitz, M., et al. (2021). NMD inhibition by 5-azacytidine augments presentation of immunogenic frameshift-derived neoepitopes. iScience *24*, 102389. https://doi.org/10.1016/j.isci.2021.102389.

11. Palma, M., and Lejeune, F. (2021). Deciphering the molecular mechanism of stop codon readthrough. Biol. Rev. Camb. Philos. Soc. *96*, 310–329. https://doi.org/10.1111/brv.12657.

12. Martins-Dias, P., and Romão, L. (2021). Nonsense suppression therapies in human genetic diseases. Cell. Mol. Life Sci. *78*, 4677–4701. https://doi.org/10.1007/s00018-021-03809-7.

13. Leroy, C., Spelier, S., Essonghe, N.C., Poix, V., Kong, R., Gizzi, P., Bourban, C., Amand, S., Bailly, C., Guilbert, R., et al. (2023). Use of 2,6-diaminopurine as a potent suppressor of UGA premature stop codons in cystic fibrosis. Mol. Ther. *31*, 970–985. https://doi.org/10.1016/j.ymthe.2023.01.014.

14. Trzaska, C., Amand, S., Bailly, C., Leroy, C., Marchand, V., Duvernois-Berthet, E., Saliou, J.M., Benhabiles, H., Werkmeister, E., Chassat, T., et al. (2020). 2,6-Diaminopurine as a highly potent corrector of UGA nonsense mutations. Nat. Commun. *11*, 1509. https://doi.org/10.1038/s41467-020-15140-z.

15. Michicich, M., Traylor, Z., McCoy, C., Valerio, D.M., Wilson, A., Schneider, M., Davis, S., Barabas, A., Mann, R.J., LePage, D.F., et al. (2025). A W1282X cystic fibrosis mouse allows the study of pharmacological and gene-editing therapeutics to restore CFTR function. J. Cyst. Fibros. *24*, 164–174. https://doi.org/10.1016/j.jcf.2024.10.008.

16. Toledano, I., Supek, F., and Lehner, B. (2024). Genome-scale quantification and prediction of pathogenic stop codon readthrough by small molecules. Nat. Genet. *56*, 1914–1924. https://doi.org/10.1038/s41588-024-01878-5.

17. Lucey, B.P., Nelson-Rees, W.A., and Hutchins, G.M. (2009). Henrietta Lacks, HeLa cells, and cell culture contamination. Arch. Pathol. Lab Med. *133*, 1463–1467. https://doi.org/10.5858/133.9.1463.

18. Reglero-Real, N., Colom, B., Bodkin, J.V., and Nourshargh, S. (2016). Endothelial Cell Junctional Adhesion Molecules: Role and Regulation of Expression in Inflammation. Arterioscler. Thromb. Vasc. Biol. *36*, 2048–2057. https://doi.org/10.1161/ATVBAHA.116.307610.

19. Delannoy, E., Tellier, G., Cholet, J., Leroy, A.M., Treizebré, A., and Soncin, F. (2022). Multi-Layered Human Blood Vessels-on-Chip Design Using Double Viscous Finger Patterning. Biomedicines *10*, 797. https://doi.org/10.3390/biomedicines10040797.

20. Apcher, S., Daskalogianni, C., Lejeune, F., Manoury, B., Imhoos, G., Heslop, L., and Fåhraeus, R. (2011). Major source of antigenic peptides for the MHC class I pathway is produced during the pioneer round of mRNA translation. Proc. Natl. Acad. Sci. USA *108*, 11572–11577. https://doi.org/10.1073/pnas.1104104108.

21. Goodenough, E., Robinson, T.M., Zook, M.B., Flanigan, K.M., Atkins, J.F., Howard, M.T., and Eisenlohr, L.C. (2014). Cryptic MHC class I-binding peptides are revealed by aminoglycoside-induced stop codon read-through into the 3' UTR. Proc. Natl. Acad. Sci. USA *111*, 5670–5675. https://doi.org/10.1073/pnas.1402670111.

22. Benhabiles, H., Gonzalez-Hilarion, S., Amand, S., Bailly, C., Prévotat, A., Reix, P., Hubert, D., Adriaenssens, E., Rebuffat, S., Tulasne, D., and Lejeune, F. (2017). Optimized approach for the identification of highly efficient correctors of nonsense mutations in human diseases. PLoS One *12*, e0187930. https://doi.org/10.1371/journal.pone.0187930.

23. Porgador, A., Yewdell, J.W., Deng, Y., Bennink, J.R., and Germain, R.N. (1997). Localization, quantitation, and *in situ* detection of specific peptide-MHC class I complexes using a monoclonal antibody. Immunity *6*, 715–726. https://doi.org/10.1016/s1074-7613(00)80447-1.

24. Zajonc, D.M. (2020). Unconventional Peptide Presentation by Classical MHC Class I and Implications for T and NK Cell Activation. Int. J. Mol. Sci. *21*, 7561. https://doi.org/10.3390/ijms21207561.

25. Huldani, H., Abdul-Jabbar Ali, S., Al-Dolaimy, F., Hjazi, A., Denis Andreevich, N., Oudaha, K.H., Almulla, A.F., Alsaalamy, A., Kareem Oudah, S., and Mustafa, Y.F. (2023). The potential role of interleukins and interferons in ovarian cancer. Cytokine *171*, 156379. https://doi.org/10.1016/j.cyto.2023.156379.

26. Gu, Q., Qi, A., Wang, N., Zhou, Z., and Zhou, X. (2024). Macrophage dynamics in prostate cancer: Molecular to therapeutic insights. Biomed. Pharmacother. *177*, 117002. https://doi.org/10.1016/j.biopha.2024.117002.

27. Chew, V., Toh, H.C., and Abastado, J.P. (2012). Immune microenvironment in tumor progression: characteristics and challenges for therapy. J. Oncol. *2012*, 608406. https://doi.org/10.1155/2012/608406.

28. Yi, M., Li, T., Niu, M., Mei, Q., Zhao, B., Chu, Q., Dai, Z., and Wu, K. (2023). Exploiting innate immunity for cancer immunotherapy. Mol. Cancer *22*, 187. https://doi.org/10.1186/s12943-023-01885-w.

29. Giles, J.R., Globig, A.M., Kaech, S.M., and Wherry, E.J. (2023). CD8(+) T cells in the cancer-immunity cycle. Immunity *56*, 2231–2253. https://doi.org/10.1016/j.immuni.2023.09.005.

30. Kumar, S., Singh, S.K., Rana, B., and Rana, A. (2021). Tumor-infiltrating CD8(+) T cell antitumor efficacy and exhaustion: molecular insights. Drug Discov. Today *26*, 951–967. https://doi.org/10.1016/j.drudis.2021.01.002.

31. Radoja, S., Saio, M., Schaer, D., Koneru, M., Vukmanovic, S., and Frey, A.B. (2001). CD8(+) tumor-infiltrating T cells are deficient in perforin-mediated cytolytic activity due to defective microtubule-organizing center mobilization and lytic granule exocytosis. J. Immunol. *167*, 5042–5051. https://doi.org/10.4049/jimmunol.167.9.5042.

32. Yu, P., and Fu, Y.X. (2006). Tumor-infiltrating T lymphocytes: friends or foes? Lab. Invest. *86*, 231–245. https://doi.org/10.1038/labinvest.3700389.

33. Young, H.A., and Hardy, K.J. (1995). Role of interferon-gamma in immune cell regulation. J. Leukoc. Biol. *58*, 373–381.

34. Jongsma, M.L.M., Guarda, G., and Spaapen, R.M. (2019). The regulatory network behind MHC class I expression. Mol. Immunol. *113*, 16–21. https://doi.org/10.1016/j.molimm.2017.12.005.

35. Ni, L., and Lu, J. (2018). Interferon gamma in cancer immunotherapy. Cancer Med. *7*, 4509–4516. https://doi.org/10.1002/cam4.1700.

36. Vigneron, N. (2015). Human Tumor Antigens and Cancer Immunotherapy. Biomed. Res. Int. *2015*, 948501. https://doi.org/10.1155/2015/948501.

37. Gocher, A.M., Workman, C.J., and Vignali, D.A.A. (2022). Interferon-gamma: teammate or opponent in the tumour microenvironment? Nat. Rev. Immunol. *22*, 158–172. https://doi.org/10.1038/s41577-021-00566-3.

38. Jorgovanovic, D., Song, M., Wang, L., and Zhang, Y. (2020). Roles of IFN-gamma in tumor progression and regression: a review. Biomark. Res. *8*, 49. https://doi.org/10.1186/s40364-020-00228-x.

39. Martini, M., Testi, M.G., Pasetto, M., Picchio, M.C., Innamorati, G., Mazzocco, M., Ugel, S., Cingarlini, S., Bronte, V., Zanovello, P., et al. (2010). IFN-gamma-mediated upmodulation of MHC class I expression activates tumor-specific immune response in a mouse model of prostate cancer. Vaccine *28*, 3548–3557. https://doi.org/10.1016/j.vaccine.2010.03.007.

40. Zhang, C., Hou, D., Wei, H., Zhao, M., Yang, L., Liu, Q., Zhang, X., Gong, Y., and Shao, C. (2016). Lack of interferon-gamma receptor results in a microenvironment favorable for intestinal tumorigenesis. Oncotarget *7*, 42099–42109. https://doi.org/10.18632/oncotarget.9867.

41. Zhu, S., Bai, Y., Zhang, D., Huang, C., Qian, X., Li, P., Xie, T., Wu, Q., Liu, A., Zhu, T., et al. (2025). Inhibiting UPF1 methylation enhances tumor immunotherapy sensitivity by reducing nonsense-mediated mRNA decay. Cell Rep. *44*, 115919. https://doi.org/10.1016/j.celrep.2025.115919.

42. Le Faouder, J., Arnaud, B., Lavigne, R., Lucas, C., Com, E., Bouvret, E., Dinel, A.L., and Pineau, C. (2022). Fish Hydrolysate Supplementation Prevents Stress-Induced Dysregulation of Hippocampal Proteins Relative to Mitochondrial Metabolism and the Neuronal Network in Mice. Foods *11*, 1591. https://doi.org/10.3390/foods11111591.

YMTHE, Volume *33*

Supplemental Information

Exploring the role of readthrough-inducing molecule 2,6-diaminopurine to increase immune response against cancer cells

Carmen Sandoval Pacheco, Alice M. Leroy, Mehdi Derhourhi, Tristan Cardon, Catherine Leroy, Nathalie Jouy, Emmanuelle Com, Blandine Guevel, Roland Bourette, Julie Carrard, Daniela Barros, Belinda Duchêne, Bénédicte Toussaint, Philippe Froguel, Nicolas Jonckheere, Thierry Chassat, Isabelle Van Seuningen, Régis Lavigne, Charles Pineau, Philippe Pierre, Fabrice Soncin, Michel Salzet, Amélie Bonnefond, and Fabrice Lejeune

Supplemental information

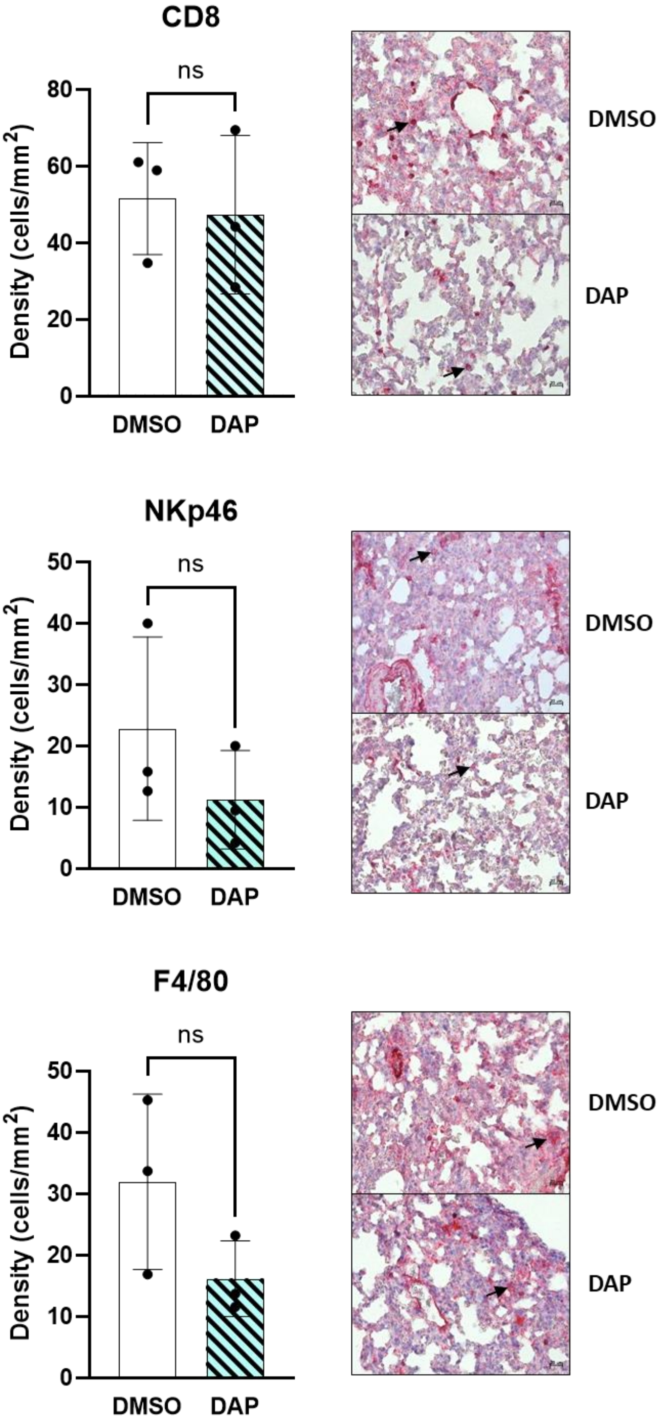

Figure S1: immunohistochemistry analysis of lymphocytes CD8+ (CD8, upper), natural killer (middle, NKp46) and macrophage (lower, F4/80) cells in healthy lung tissue after mouse treatment with DMSO or DAP. On the left is shown the quantification from 3 tumor slides. One representative picture of the analysis is presented on the right. Black arrows indicate the expression of interest. P-values were calculated by T-Test: n.s: statistically not significant.

A

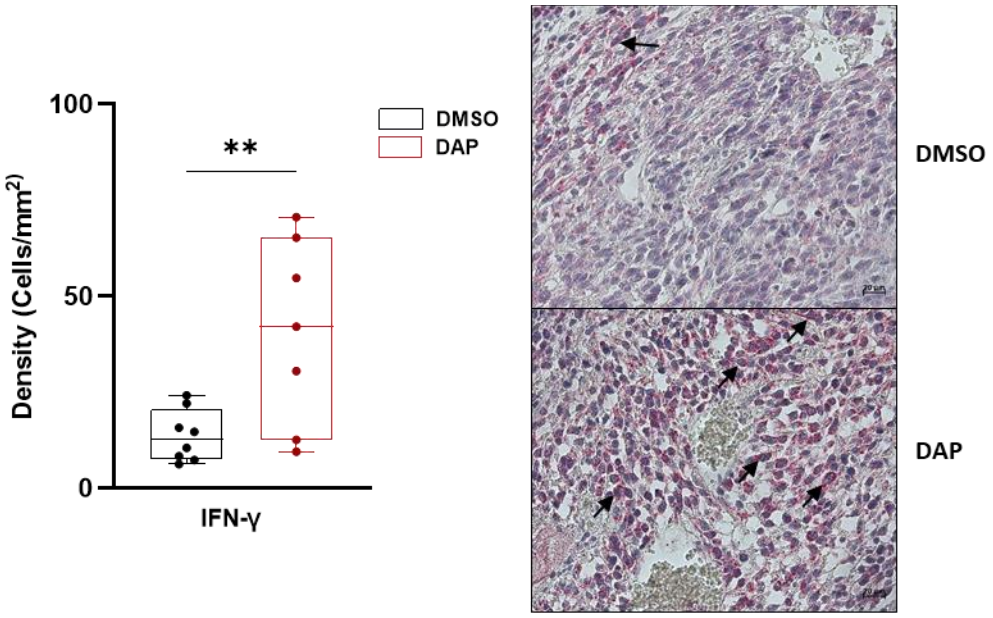

B

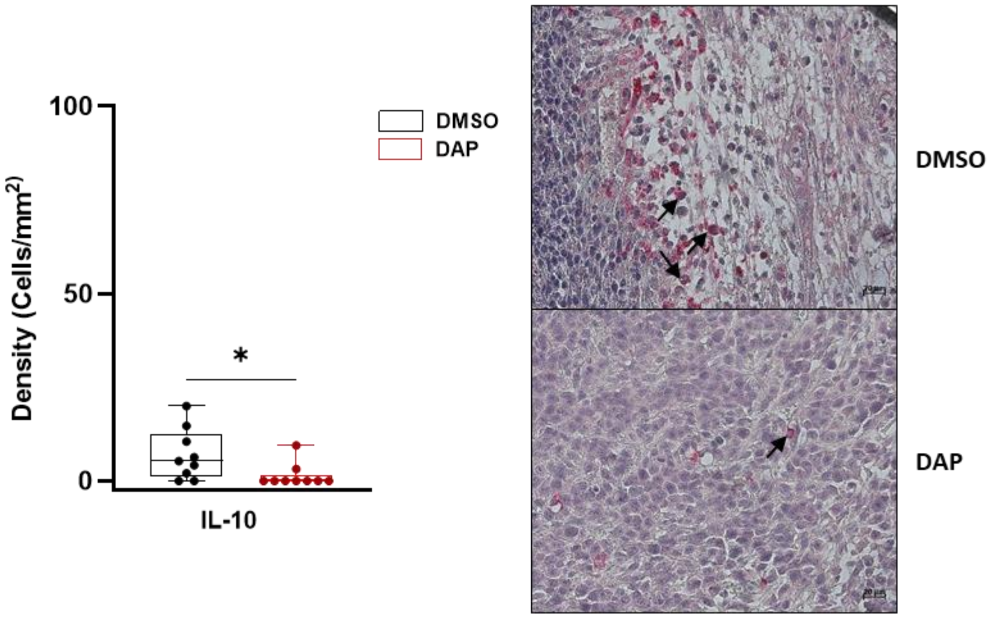

Figure S2: Immunohistochemistry analysis of IFN-γ expression (upper panel) and IL-10 expression (lower panel) in mice treated with DMSO or DAP. On the left is shown the quantification from 10 tumor slides. One representative picture of the analysis is presented on the right. Black arrows indicate the expression of interest. P-values were calculated by T-Test *<0.05; **<0.01.

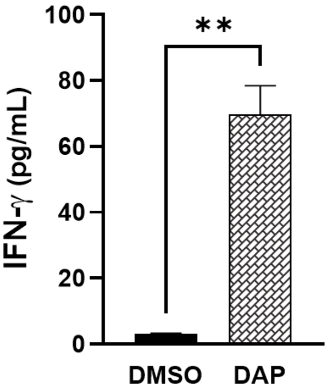

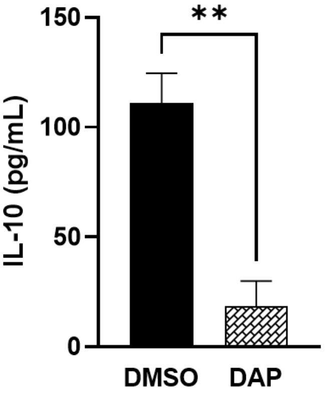

Figure S3: Quantification of IFN-γ and IL-10 expression by ELISA in co-cultures of CT26 cells and white blood cells (WBCs) isolated from the spleen of an untreated Balb/C mouse. WBCs and CT26 cells were co-cultured for 24 hours in the presence of anti-CD3 and anti-CD28 antibodies and either DMSO or DAP, followed by ELISA-based measurement of IFN-γ and IL-10 levels. P-values were calculated using a T-test **p < 0.01. The results shown are representative of two independent experiments.

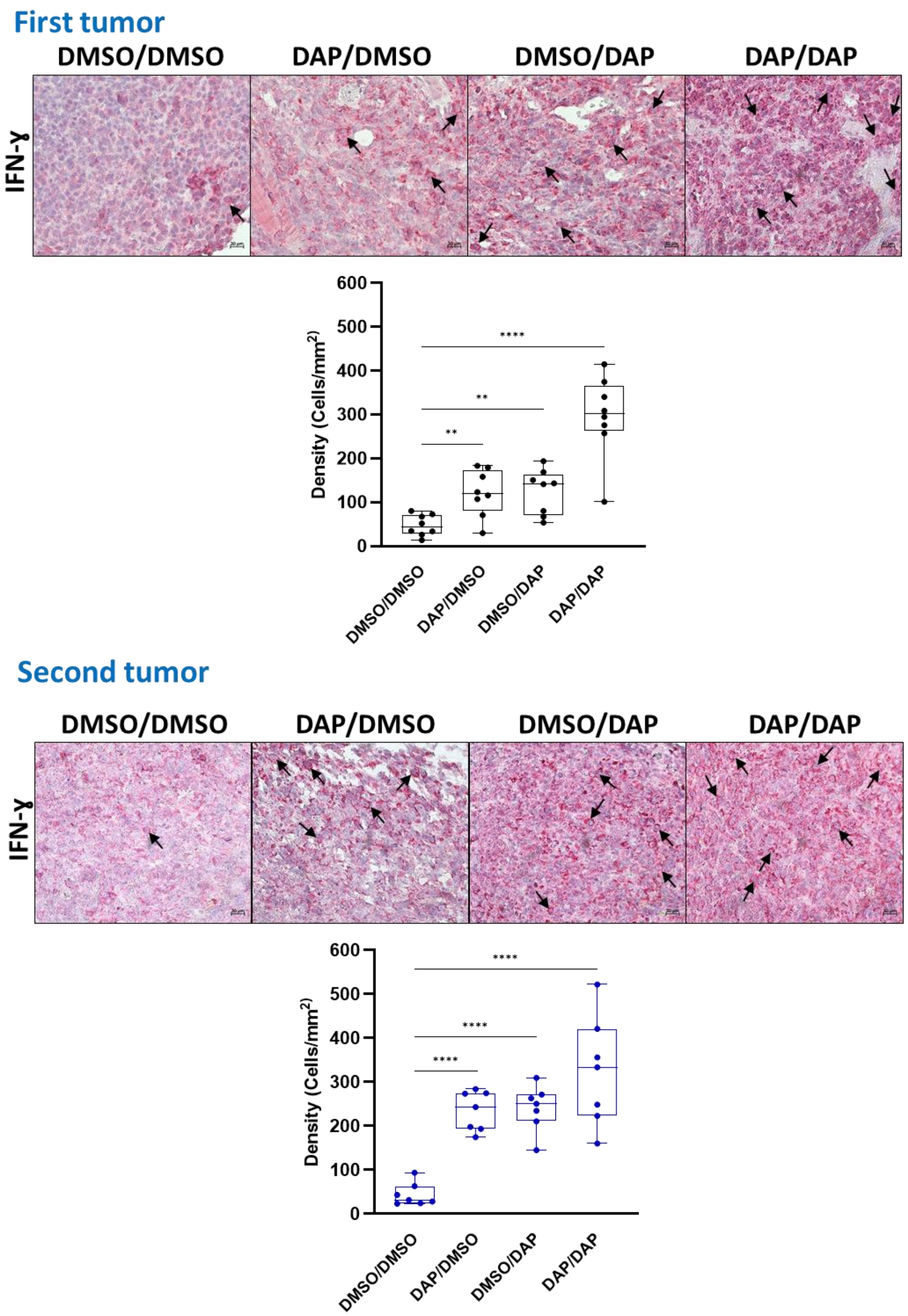

Figure S4: immunohistochemistry analysis of IFN-γ expression in first tumors (upper panel) and second tumors (lower panel) issued from cells treated with DMSO or 100 µM DAP. Quantification is based on the analysis of 10 pictures. One representative picture of the analysis is presented on the right. The

black arrows indicate the expression of interest. P-values were calculated by T-Test *<0.05; **<0.01; ***<0.001; ****<0.0001.

First Tumor

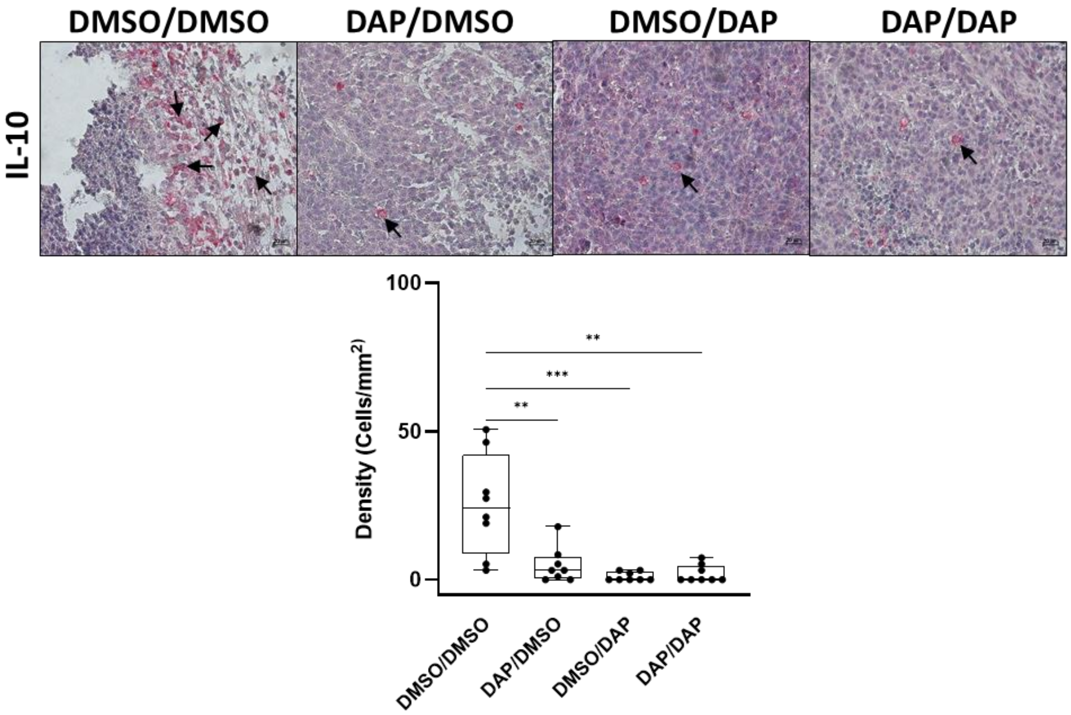

Second Tumor

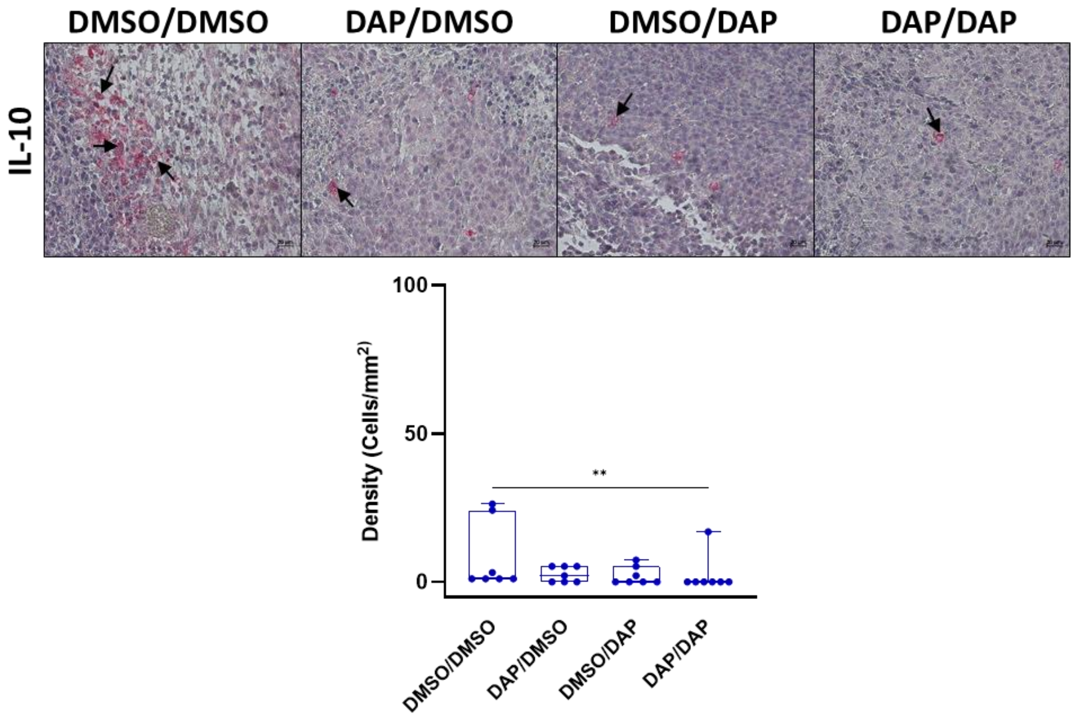

Figure S5: immunohistochemistry analysis of interleukin 10 expression in first tumors (upper panel) and second tumors (lower panel) issued from cells treated with DMSO or 100 µM DAP. Quantification

is based on the analysis of 10 pictures. One representative picture of the analysis is presented on the right. Black arrows indicate the expression of interest. P-values were calculated by T-Test *<0.05; **<0.01; ***<0.001; ****<0.0001.

Table S1: list of proteins that have their expression modified in the presence of DAP compared to DMSO treatment.

Supplemental method

Isolation of White Blood Cells (WBC) from Mouse Spleen

Splenic white blood cells were collected from BALB/c mice. We used a 70 µm cell strainer for mechanical dissociation into cold PBS. Red blood cells were removed using hypotonic in-house lysis buffer containing 155 mM NH_4Cl, 10 mM $KHCO_3$, and 0.1 mM EDTA at pH 7.4. After a 5-minute incubation, the cells were washed with 10 mL of PBS and then resuspended in complete RPMI medium containing 10% fetal bovine serum (Sigma-Aldrich) and 1% zellshield (Minerva Biolabs).

In vitro CT26 cells and WBC co-culture assay

CT26 cells were seeded in 24-well plates at a density of 1×10^5 cells per well and allowed to adhere overnight. Cells were then treated for 24 hours with either DMSO or 25µM DAP. Following the treatment, WBCs were added at a 5:1 ratio (WBC /tumor cell). T cell activation was performed by adding anti-CD3 (ref 16-0032-82, Invitrogen) and anti-CD28 (ref 16-0281-82, Invitrogen) at a 1:3 ratio. Cocultures were incubated at 37°C with 5% CO_2 for 24 hours. The supernatants were harvested, and the IL-10 and IFN-γ levels were measured.

ELISA

Concentrations of IFN-ɣ and IL-10 in cell culture supernatants were determined using commercially available ELISA kits for mouse cytokines (BMS606-2 and BMS614, Invitrogen), respectively, according to the manufacturer’s instructions. Absorbance was measured at 450 nm.
